# Population Genetic Patterns of Threatened European Mudminnow (*Umbra krameri* Walbaum, 1792) in a Fragmented Landscape: Implications for Conservation Management

**DOI:** 10.1371/journal.pone.0138640

**Published:** 2015-09-22

**Authors:** Péter Takács, Tibor Erős, András Specziár, Péter Sály, Zoltán Vitál, Árpád Ferincz, Tamás Molnár, Zoltán Szabolcsi, Péter Bíró, Eszter Csoma

**Affiliations:** 1 Balaton Limnological Institute, Centre for Ecological Research, MTA, Tihany, Hungary; 2 Department of Aquaculture, Szent István University, Gödöllő, Hungary; 3 Department of Nature Conservation, Kaposvár University, Kaposvár, Hungary; 4 Institute of Forensic Medicine, Network of Forensic Science Institutes, Budapest, Hungary; 5 Department of Medical Microbiology, University of Debrecen, Debrecen, Hungary; University of California Santa Cruz, UNITED STATES

## Abstract

The European mudminnow (*Umbra krameri*) is a Middle Danubian endemic fish species, which is characterised by isolated populations living mainly in artificial habitats in the centre of its range, in the Carpathian Basin. For their long term preservation, reliable information is needed about the structure of stocks and the level of isolation. The recent distribution pattern, and the population genetic structure within and among regions were investigated to designate the Evolutionary Significant, Conservation and Management Units (ESUs, CUs, MUs) and to explore the conservation biological value of the shrinking populations. In total, eight microsatellite loci were studied in 404 specimens originating from eight regions. The results revealed a pronounced population structure, where strictly limited gene flow was detected among regions, as well as various strengths of connections within regions. Following the results of hierarchical structure analyses, two ESUs were supposed in the Carpathian Basin, corresponding to the Danube and Tisza catchments. Our results recommend designating the borders of CUs in an 80–90km range and 16 clusters should be set up as MUs for the 33 investigated populations. How these genetic findings can be used to better allocate conservation resources for the long term maintenance of the metapopulation structure of this threathened endemic fish is discussed.

## Introduction

Although large floodplain rivers and their associated habitats (e.g., dead arms, backwaters, wetlands) represent a small fraction of the total land area, they nonetheless harbor an outstanding number of endangered and threatened species worldwide [1, 2]. However, large rivers in the developed regions of the world are highly influenced by anthropogenic impacts [3], [4] such as river regulation, construction of flood-control levees, and reservoirs, which disrupt the lateral and longitudinal connections of aquatic systems [5, 6]. Moreover, growing human populations, increased agricultural land use, and the development of infrastructure decreases and fragments the area of natural floodplain ecosystems [7].

Habitat degradation results in the erosion of genetic diversity [8, 9], in particular for those aquatic species which are characterised by special environmental needs, narrow ranges of distribution, and low dispersal ability [10, 11]. Therefore for certain conservation biological projects, such as habitat rehabilitation and reconstruction, the knowledge of population genetic structure is necessary if, for example, resettlement of these species is planned [12, 13]. The task of population genetic investigations in such cases is to assess the rate of gene flow among populations, to identify shrunken populations exposed to the adverse effects of inbreeding, and particularly to determine evolutionary significant units (ESU) within biogeographical patterns [14–17]. This information can be used directly to elaborate species conservation and management plans [18, 19].

**Fig 1:**
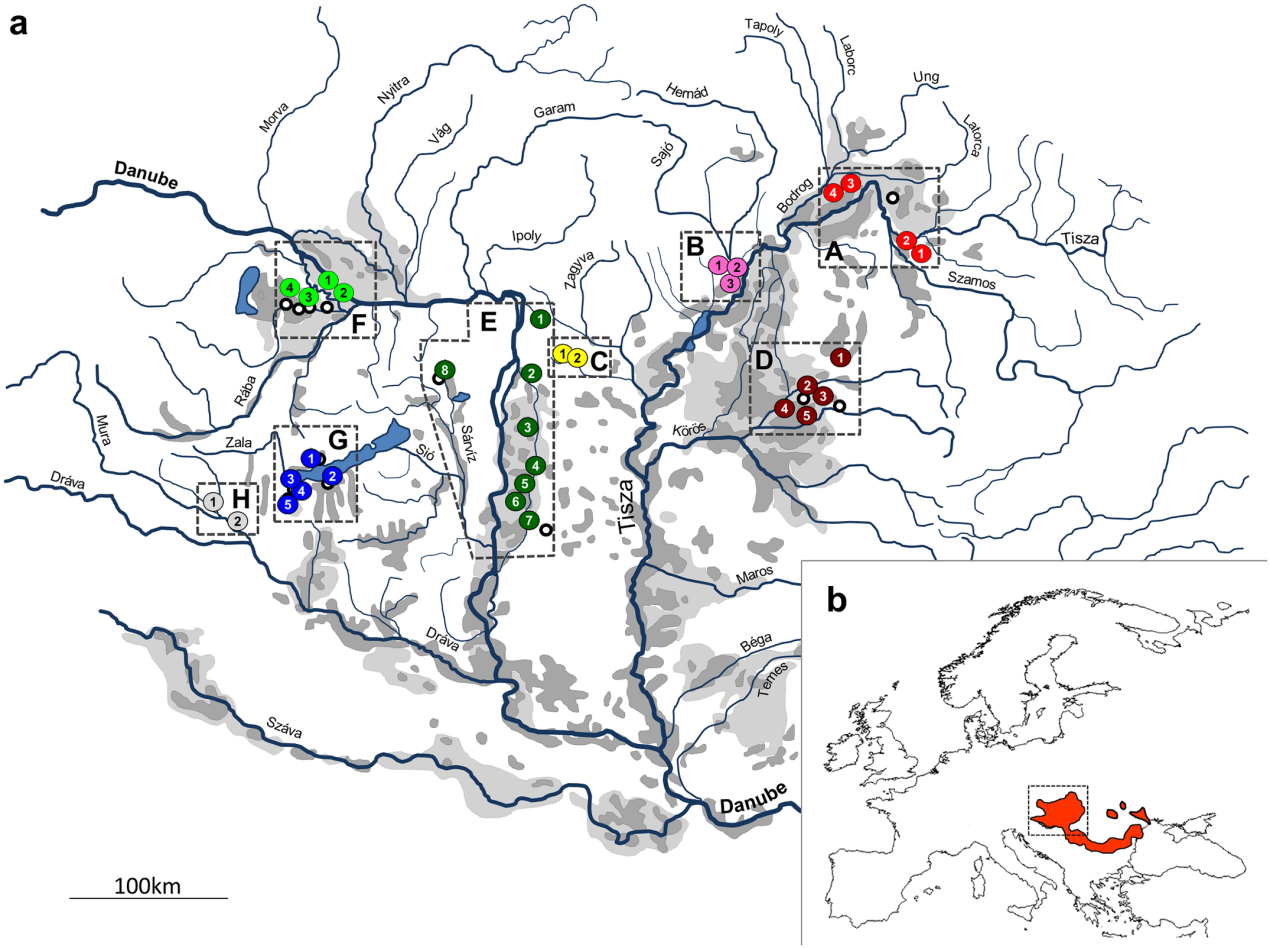
(a) Recent river network of the Carpathian Basin, with 33 sampling sites across eight sampling regions. Sites indicated by different colours belong to different regions. Open circles indicate further known (but not analysed) stocks of mudminnow. Periodically and permanently flooded areas before the beginning of river regulation works (in the mid. 19th century) are indicated by light and dark grey patches respectively. For detailed information, refer to the text and Table 1. (b) Distribution area of European muddminow (red coloured area). The location of the Carpathian Basin in Europe is indicated by a dotted rectangle.

In Europe, up to 90% of the floodplain area is altered from its natural state [20]. Although less well known, the most severe degradation of floodplain systems has occurred in the Carpathian Basin. Until the middle of the 19th century, more than 21,000 km^2^ of land was flooded at least periodically in the basin [21]. However, of the ~15,000 km^2^ floodplain area of the Tisza River (the largest tributary of Danube River, with a drainage area of 157,000 km^2^), for example, approximately 4,400 km^2^ was permanently inundated [22] (see Fig 1a). River regulation began in the middle of the 19th century and more than 4,200 kms of levees have since been built, and hundreds of larger meanders were cut along the river. Therefore, the course of the Tisza River was shortened from the original 1419 to 966 km (i.e., by 32%) [20]. The length of the Berettyó River, a tributary of the Tisza River, was also shortened, to half of the original length (from 364 to 174 km) [23]. Concurrent with river regulation, draining of the marshlands was also carried out. The length of draining canals is currently greater than 40,000 km in the central area of the Carpathian Basin [24]. As the result of these large-scale and numerous regulations, the wetland area in the floodplain of the Tisza River was reduced to 539 km^2^. In the entire Carpathian Basin, the area of wetlands and backwaters directly connected to rivers was reduced to 700 km^2^ [21]. This broad alteration of the environment has consequently had a serious effect on the fauna. Several vertebrate species preferring these aquatic habitats became rare or extirpated from the basin [25], including some endemic fish species, such as the European mudminnow (*Umbra krameri* Walbaum, 1792), a Middle Danubian endemic fish species [26], which has suffered a serious population decline. This small (up to 100 mm in total length) stagnophilous species [27] was widely distributed in the marshy habitats of the Danube drainage basin from Vienna to the delta, and in the lower reaches of Dniestr drainage basin [28], prior to river regulation (Fig 1b). The centre of its distribution area is the inner Carpathian Basin [29, 30], where this species was so abundant, that it was used to feed domestic animals (e.g., swine) [31]. At present, only a few sporadic populations have survived the habitat loss and fragmentation [32, 33]. The unexpected invasion of a highly competitive, non-native fish species, the Amur sleeper (*Perccottus glenii* Dybowsky 1877), has further worsened the situation, especially in the Tiszanian area [34, 35]. Unfortunately, this voracious competitor has recently also appeared in the middle and western parts of the Carpathian Basin [36–38]. Due to these impacts, mudminnow is known to have been extirpated from many locations [32], especially from the backwaters connected to the Hungarian Upper and Middle Tisza sections. In just the last decades, more than 30% population decline is estimated to have occurred [28], and European mudminnow has therefore been listed as vulnerable (VU) on the IUCN Red List [39] since 1996. Although the mudminnow is a strictly protected species in Hungary, only few studies have carried out a comprehensive analysis of the distribution of the mudminnow, and are in need of a more recent update [32]. In order to promote the conservation of the remaining stock, captive breeding and rearing techniques have been developed [40, 41], along with some pilot habitat revitalization and population translocation (transferring residual stocks from threatened habitats to newly established nature-like habitats) experiments [42, 43]. Nevertheless, we still do not have a comprehensive overview of the genetic structure of the remaining populations distributed across shrinking and strongly fragmented habitat patches in the Carpathian Basin. According to the findings of field surveys, many of the known populations now consist only of very few individuals, which indicates a high probability of inbreeding and the extreme risk of their extirpation [32, 42, 44]. We do not know, however, how the total genetic diversity of the mudminnow is distributed among populations. Regarding the rapid decrease of mudminnow populations and the limited amount of resources available for their conservation, it is especially important to explore population genetic patterns across the distribution area (i.e., toward the identification of ESUs), and to identify those populations containing most of the genetic diversity in order to set priorities for an effective action plan. For instance, imperative species conservation actions strongly demand information on which populations may be used for supplementing (i.e., serving as a pool of parent fish in artificial breeding programs) endangered populations and stocking reconstructed habitats in different geographic areas and on which populations have the genetic uniquity requiring them to be preserved without mixing them with other populations (i.e., the determination of conservation and management units, MUs and CUs) [17, 45].

Therefore, the aims of the current study were: (i) to explore the recent distribution of mudminnow populations in the Hungarian part of the Carpathian Basin, the area inhabited by the majority of the known stocks; (ii) to reveal spatial patterns in the population genetic structure and among sites’ migratory rates; (iii) to assess the conservation biological value of extremely small populations; and (iv) to designate ESUs, CUs and MUs.

## Materials and Methods

### Ethics Statement

This study was carried out following relevant national and international guidelines pertaining to the care and welfare of fish. Our surveys (e.g., tissue sampling during field surveys) are not qualified as animal tests by the operative Hungarian law (N^o^: 40/2013.(II.14.). However, since the studied species is strictly protected in Hungary, any procedure to be applied in connection with it is controlled by the order of government N^o^: 348/2006.(XII.23.), and is subject to authorisation from the National Inspectorate for Environment, Nature and Water. The collection and storage of mudminnow tissue samples was authorized by this bureau (permission numbers: 14/881/5/2011, 14/678-9/2012). During the sampling procedure, all efforts were made to minimize suffering. Field studies did not involve other fish species that were endangered according to the IUCN Red List of Threatened Species v. 2014.3 (www.iucnredlist.org).

### Sample collection, DNA extraction and microsatellite amplification

Studied areas and sites were selected based on the last published distribution data of mudminnow [32]. Specimens were collected by electrofishing between 2011 and 2013, from each region of the Carpathian Basin where the presence of this species had previously been noted (Table 1). To minimize suffering, individuals collected for this study (N = 5–20 per site, depending on the stock size) were narcotized using clove oil. The active agent of clove oil is eugenol (4-allyl-2-ethoxyphenol), which is considered non-carcinogenic, non-mutagenic, and a "Generally Recognized As Safe" (GRAS) substance by the U.S. Food and Drug Administration [46, 47]. The aqueous emulsion of clove oil was deposited into the dangerous refuse containers of the Balaton Limnological Institute after use. For the genetic survey, an approximately 2mm^2^ anal fin clip was sampled from each specimen and stored in 96% ethanol at -20°C until DNA extraction. After fin tissue sampling, fish were released to the habitat they were captured from. DNA was isolated with DNeasy Blood and Tissue kit (Qiagen, Germany), using 10–20 mg of tissue, as per the manufacturer’s instructions. Quality and quantity of the extracted DNA were verified using a NanoDrop 2000c Spectrophotometer (Thermo Scientific, USA).

**Table 1:** Name, location, code, and geographical position of the sampling sites.

Drainage Basin	Region	Name of river	code	HC	Coordinates	N	Ar	I	Ho	He	F	P
Tisza	Upper Tisza	Gőgő-Szenke	A1	n	N47.96629 E22.60042	14	7.25	1.63	0.76	0.73	-0.03	0.95
		Öreg-Túr	A2	n	N48.03644 E22.52039	12	6.25	1.48	0.72	0.69	-0.04	0.65
		Bélyi-csatorna	A3	a	N48.37069 E22.00521	5	4.50	1.29	0.83	0.67	-0.24	1.00
		Ricsei-csatorna	A4	a	N48.33958 E21.97172	15	7.00	1.53	0.71	0.70	-0.02	0.34
	Borsodi plain	Hejő	B1	a	N47.91033 E20.90809	15	9.00	1.83	0.80	0.75	-0.06	0.95
		Hejő	B2	a	N47.86618 E21.00431	15	10.00	1.97	0.76	0.78	0.09	0.22
		Rigós-ér	B3	a	N47.80835 E20.97225	10	5.88	1.48	0.71	0.70	-0.04	0.15
	Tápió	Felső-Tápió	C1	a	N47.38396 E19.71634	5	3.00	0.86	0.58	0.49	-0.18	0.98
		Felső-Tápió	C2	a	N47.35976 E19.75206	15	3.38	0.83	0.48	0.46	-0.05	0.99
	Bihari plain	Pocsaji-láp	D1	n	N47.30103 E21.85952	15	3.00	0.87	0.50	0.51	0.03	0.23
		Kis-Körös	D2	a	N47.21176 E21.64530	10	3.67	0.83	0.44	0.44	0.00	0.29
		Ölyvös-ér	D3	a	N47.17127 E21.72658	10	4.25	0.99	0.49	0.50	0.02	0.61
		Kutas-ér	D4	a	N47.06114 E21.46210	10	7.25	1.66	0.79	0.73	-0.08	0.35
		Csente-Szakáli-alsó-csatorna	D5	a	N47.01788 E21.59800	5	4.50	1.26	0.65	0.63	-0.02	0.45
Danube	Middle Hungarian	Sződ-Rákos-patak	E1	n	N47.62597 E19.29635	20	4.88	1.02	0.51	0.53	0.04	0.06
		Öreg-turjános	E2	n	N47.29726 E19.20590	10	5.00	1.31	0.64	0.66	0.01	0.54
		Adacsi- csatorna	E3	a	N46.93606 E19.32020	15	5.75	1.35	0.63	0.65	0.00	0.07
		Kolon-tavi-övcsatorna	E4	a	N46.75006 E19.30508	15	7.00	1.53	0.76	0.70	-0.10	0.62
		Szölőaljai-csatorna	E5	a	N46.64870 E19.23308	5	4.38	1.20	0.73	0.63	-0.17	0.99
		Székesi-csatorna	E6	a	N46.50555 E19.05487	12	7.25	1.69	0.79	0.77	-0.02	0.81
		Karasica-főcsatorna	E7	a	N46.44800 E19.10021	15	7.13	1.61	0.79	0.74	-0.07	0.73
		Császárvíz-övárok	E8	a	N47.36556 E18.48591	10	2.75	0.80	0.69	0.49	-0.36	0.02
	Hanság-Szigetköz	Örömkő-Laposai-csatorna	F1	a	N47.74231 E17.62207	15	6.88	1.54	0.69	0.70	0.00	0.49
		Bácsai-csatorna	F2	a	N47.74036 E17.65363	5	4.50	1.31	0.85	0.68	-0.25	1.00
		Lébénymiklósi-csatorna	F3	a	N47.74922 E17.36127	15	8.25	1.75	0.82	0.77	-0.08	0.43
		Bordacs-Császárréti-csatorna	F4	a	N47.78628 E17.26714	15	7.00	1.64	0.79	0.76	-0.03	0.18
	Balaton	Lesence	G1	a	N46.80347 E17.40451	15	6.00	1.37	0.69	0.65	-0.07	0.63
		Ordacsehi-berek	G2	n	N46.75207 E17.60159	15	4.38	1.05	0.52	0.53	0.04	0.01
		Kis-Balaton (navvy hole)	G3	a	N46.69515 E17.24623	15	7.25	1.52	0.68	0.67	0.00	0.89
		Marótvölgyi-vízfolyás	G4	a	N46.58907 E17.28098	15	7.25	1.69	0.79	0.76	-0.03	0.92
		Zala-Somogy-határárok	G5	a	N46.53436 E17.22207	11	7.13	1.64	0.76	0.75	-0.04	0.51
	Mura	Kerka-malomárok	H1	a	N46.51676 E16.57354	15	4.25	1.00	0.49	0.52	0.07	0.62
		Holt-Mura	H2	n	N46.38936 E16.77413	10	5.00	1.22	0.63	0.59	-0.06	0.97

HC: habitat condition (n: natural, a: artificial (modified)). N: number of specimens. Genetic diversity indices of microsatellite marker data: Ar: mean of allelic richness; I: Shannon's Information Index; Ho: observed heterozygosity; He: expected heterozygosity; F: fixation index (F = 1-(Ho He^-1^)); P: probability of the Hardy-Weinberg equilibrium test.

Nine microsatellite markers previously developed by Winkler and Weiss [48] were investigated by multiplex PCR. 50 ng of DNA was amplified in a multiplex PCR reaction with three primer pairs in a PCR reaction using Type-it Microsatellite PCR Kit (Qiagen, USA), and fluorescently labelled forward primers according to the protocol of the kit (details and multiplex combinations are provided in Table 2). The final concentration of each primer was 0.2 μM, and the annealing temperature was 60°C in each PCR. Amplified products were detected using an ABI 3130 sequencer (Applied Biosystems, USA). Peak Scanner Software v1.0 was used to analyse the data. The functionality of all primer pairs and the reliability of multiplex PCR were tested in singleplex reactions using the same PCR conditions and reagents.

**Table 2:** Primer sequences, fluorescent dyes and combinations of primers in three (1., 2., 3.) multiplex PCR, number of alleles, locus-specific F_st_ values, number of populations out of the 33 in which deviations from Hardy-Weinberg Equilibrium (HWE) were detected. (*UkrTet2 UkrTet3 sequences are identical, therefore locus UkrTet2 was omitted from the further analyses, for more details refer to text), and Mean ± SD estimated frequency of null alleles.

Multiplex PCR	primer	5’-3’ sequence of the primer		5’ fluores-cent dye	number of alleles	Locus specific *F*_*st*_ values	No. of significant (p<0.05) deviations from HWE
		forward	reverse				
1	UkrTet1	CATCAAATGTTGGCAGACTTGC	GGGAAACCGCTATCCTGAC	HEX	25	0.190	5
	UkrTet2*	AACACACAGACAGGACGTTCC	GGGAGAAAGATGGGTGCC	6-FAM	-	-	-
	UkrTet3	GGGTGCCAGGCTGTTCTC	ATCAATCGGACTAACGGTTCG	NED	19	0.148	0
2	UkrTet4	AGACGGCAGCACATAAGAA	ATTATTGGTGTCCATCCCTGTC	6-FAM	18	0.198	1
	UkrTet5	TCACCGCACAAAAGAAACAC	AACACCAGGGAACTGCAGTCT	VIC	14	0.249	2
	UkrTet7	CAATAGTTCCCCAATCCTGG	GTCTCGACCACCAAGCG	NED	14	0.482	2
3	UkrTet6	ATCGGTTTTTGCCCATCAGT	CCGCAGATCGAAAGTTTGAC	HEX	6	0.237	2
	UkrTet8	CTTGGCTGTGGTGGTTGAA	GGGGGAGTCCCTGC	6-FAM	15	0.296	1
	UkrTet9	CACAGTCTCAATGGGGGAAA	CCAAGCCTAACCTGCTAAAGA	NED	22	0.181	4

### Statistical analyses

#### Genetic variation

Microsatellite markers (Table 2) and primers published by Winkler and Weiss [48] were checked in GenBank before PCR and microsatellite analysis. Based on the available sequences, it was suggested that *Umbra krameri* microsatellites UkrTet2 (GenBank: FJ228219.1) and UkrTet3 (GenBank: FJ228220.1) sequences are identical, the reverse complements of each other. The DNA strand direction was mistakenly reversed for these. Data analysis of 156 fish samples (the first 14 populations) further strengthened this hypothesis. Allele numbers (n = 18) and allele frequencies, allele specific *F*_*st*_ values (*F*_*st*_ = 0.156) and the proportion of null alleles (prop. = 0.040372339) were equal for the UkrTet2 and UkrTet3 loci. Consequently, the UkrTet2 marker was left out of further statistical analyses.

Fisher’s exact test of linkage disequilibrium and tests for deviations from Hardy-Weinberg equilibrium were conducted using GENEPOP 4.2.2 [49] for each locus using a Markov chain of 10,000 dememorization steps, 20 batches, and with 5,000 iterations per batch. MICROCHECKER 2.2.3. [50] was used to estimate the frequency of null alleles for each locus. Number of alleles, and locus specific *F*_*st*_ values, were calculated using GenAlEx 6.5 [51]. Similarly, this software was used to estimate the mean of allelic richness (Ar), Shannon's Information Index (I), observed and expected heterozygosity (H_o_, H_e_), fixation index (F) and list the private alleles for each population.

#### Population structure

The Lynch & Ritland [52] estimator was used to calculate between-specimen pairwise relatedness. From this semimatrix, mean within-population pairwise values were calculated with 999 permutations, and 1,000 bootstraps in GenAlEx 6.5 [51]. Because in some cases null alleles were detected, their effect on pairwise *F*_*st*_ values were corrected using the ENA procedure of Chapuis and Estoup [53, 54]. Genetic distances among populations were calculated using the Cavalli-Sforza and Edwards [55] estimator after INA correction [53], and presented in PCoA ordination. GenAlEx 6.5 [51] was used to perform the analysis of molecular variance (AMOVA), with 999 permutations for population differentiation and hierarchical partitioning of genetic variation among and within regions and populations (F-statistics: *F*_*rt*_ *–*H_0_: individuals are shuffled among regions, *F*_*sr*_ *–*H_0_: individuals are shuffled within regions, *F*_*st*_ *–*H_0_: populations are shuffled among regions, *F*_*is*_–H_0_: individuals are shuffled within populations, and *F*_*it*_–H_0_: individuals are shuffled in the whole sample).

Genetic population structure was inferred using the hierarchical approach [56] of the STRUCTURE analysis [57] to estimate the most probable number of genetic groups (clusters, K) for all analysed individuals. Namely, the STRUCTURE analyses was first run including all samples, then samples were separated by river drainage basin, and finally by region. (A, B, C, etc.) Values of K were investigated from 1 to 20, with a burn-in period of 100,000 followed by 100,000 MCMC iterations and 10 runs for each K using an admixture model with correlated allele frequencies. Results of these Bayesian statistics were evaluated by STRUCTURE HARVESTER [58], implementing the (deltaK) Evanno method [59]. Results of the 10 repetitions were combined using the software CLUMPP 1.1.2. [60]. For the genetic assignment of the studied individuals, Bayesian cross validation tests [61] were carried out on drainage basin, regional, and population levels using GeneClass2 [62] software. Cross validation tests were carried out similarly on the clusters defined at various levels by the hierarchical STRUCTURE analyses.

#### Migration and spatial analyses

Migration rate estimation was performed by MIGRATE-N 3.2.15 [63, 64], using the maximum likelihood method under the following parameterization: 20 short chains (500 trees used out of the sampled 10,000) and 5 long chains (5,000 trees used out of the sampled 100,000). Missing data were not included. Theta values were generated from the *F*_*st*_-calculation. Mutation rates among loci were estimated from the data. Migration was estimated for the original regional groups designated by catchment basin, and based on the results of hierarchical STRUCTURE analyses at the three levels.

Spatial genetic structure was assessed at the population level using Mantel tests [65], through the comparison of pairwise *F*_*st*_ data and pairwise straight line geographic distances (GGD) with 999 randomisations for the whole dataset and at drainage basin levels. On the individual level, two kinds of spatial autocorrelation [66, 67] computation were made. Spatial autocorrelation computation is suitable to accurately identify the scale at which population genetic structure is detectable. In this case, “r” coefficients were calculated using multiple distance class analyses. This method plots “r” as a function of increasing distance class sizes. The last distance class for which “r” is significant is considered to be the limit of detectable isolation by distance (IBD). In the second case, autocorrelation coefficients, “R”, were plotted as a function of discrete distance classes, partitioned so as to achieve a similar number of pairwise comparisons for each class [68]. Positive and significant “R” values indicate IBD and the “x” intercept provides an estimate of the extent of IBD. For more details see Peakall et al. [69]. All of the spatial autocorrelation computations, including the GGD semimatrix calculation from geocoordinates, were made in GenAlEx v6.5 [51].

## Results and Discussion

### Results

#### Distribution patterns

As a result of our broad field investigations, European mudminnow was observed at more than 40 sampling sites from the Middle Danubian catchment (Fig 1). A total of 404 mudminnow specimens were sampled for population genetic research, from 33 sites, originating mainly from artificial habitats (i.e., ditches, canals, and ponds), across eight regions (Fig 1, Table 1). In several locations, no more than 5 individuals were able to be collected at one time, despite the intensive sampling effort.

#### Data quality and Genetic variation

Results of Fisher’s exact tests did not reveal evidence for large allele drop-out for any locus, or for linkage disequilibrium between any pairs of loci. All microsatellite loci showed significant deviation from the Hardy-Weinberg equilibrium state if all 404 specimens were analysed as one entire population (404 samples), and we calculated high *F*_*is*_ values (>0.08) at all loci among the 404 samples. These results suggest strong genetic substructure caused by geographical isolation and/or non-random mating in subgroups. Characteristics of each microsatellite locus used in our work are provided in Table 2. The total number of alleles per locus varied between 6 and 25 (mean: 17.6). Locus specific *F*_*st*_ values ranged between 0.148 and 0.482. Chi-Square Tests for Hardy-Weinberg Equilibrium for each loci at each population showed significant values in 17 cases (see Table 2 and S1 Table).

A total of 136 alleles were observed for the eight loci used in the analysis. The whole raw dataset used for further analyses is indicated in S2 Table in GENEPOP format. Average allelic richness, such as observed heterozygosity, showed high-level differences among populations. These ranged from 2.75 to 10 and from 0.44 to 0.85 respectively, within the studied populations. Shannon's Information Index showed a high level of variability as well, ranging between 0.8 and 1.97 (Table 1). Deviation from HWE was significant (p<0.05) in two studied populations (E8, G2).

As MICROCHECKER analyses proved the presence of false homozygotes (null alleles) in some cases (i.e., for UkrTet1 at sites B3, D2, E3, F1, for UkrTet3 at site E3, for UkrTet4 at site B2), therefore null allele correction was made prior to further analyses (see S3 Table).

13 of the detected 136 alleles were unique to a single location (Table 3). The frequency of the unique alleles ranged between 0.031 and 0.071. Eight of the unique alleles were observed in sites of the Tisza River drainage basin, and five from the Danubian part of the study area. No unique alleles were found for small stocks (where N = 5), in populations A3, C1, E5 and F2.

**Table 3:** Summary of private alleles (allele sizes and frequencies) by population. Codes of populations correspond to those of Table 1 and Fig 1.

Pop	Locus	Allele size	Freq.
A1	UkrTet4	185	0.036
A1	UkrTet4	189	0.071
A1	UkrTet4	193	0.036
A1	UkrTet4	197	0.036
B1	UkrTet4	173	0.033
B2	UkrTet8	226	0.033
D4	UkrTet6	224	0.050
E1	UkrTet1	211	0.025
E6	UkrTet5	314	0.042
E7	UkrTet9	317	0.033
F4	UkrTet6	244	0.067
G3	UkrTet3	243	0.033
G4	UkrTet7	204	0.033

Pairwise genetic distances ranged between 0.198 and 0.821 (mean: 0.621). Pairwise *F*_*st*_ values ranged between -0.01 and 0.476 (mean: 0.195), and only five of the 528 pairwise comparisons (A3-4, B1-2, C1-2, F1-2 and G4-5) did not show significant differences (Table 4).

**Table 4:** Pairwise F_st_ values generated by FREENA for each pair of sites using ENA method (below the diagonal). Italicized numbers indicate significant differentiations (p<0.05). Cavalli-Sforza and Edwards genetic distances for each pair of populations are indicated above the diagonal. Codes of sites correspond to those of Table 1.

	A1	A2	A3	A4	B1	B2	B3	C1	C2	D1	D2	D3	D4	D5	E1	E2	E3	E4	E5	E6	E7	E8	F1	F2	F3	F4	G1	G2	G3	G4	G5	H1	H2
A1	-	0.*413*	0.522	0.535	0.475	0.457	0.512	0.727	0.728	0.669	0.642	0.624	0.541	0.636	0.688	0.687	0.658	0.665	0.729	0.635	0.642	0.733	0.624	0.674	0.596	0.613	0.637	0.676	0.572	0.598	0.621	0.675	0.629
A2	*0*.*062*	-	0.578	0.533	0.450	0.509	0.565	0.698	0.711	0.688	0.666	0.654	0.587	0.648	0.614	0.756	0.661	0.671	0.690	0.648	0.637	0.782	0.607	0.676	0.554	0.587	0.630	0.637	0.567	0.622	0.630	0.680	0.649
A3	*0*.*078*	*0*.*095*	-	0.384	0.515	0.530	0.512	0.711	0.698	0.601	0.545	0.551	0.593	0.665	0.675	0.764	0.721	0.701	0.732	0.652	0.697	0.777	0.669	0.669	0.628	0.643	0.626	0.693	0.597	0.655	0.648	0.716	0.682
A4	*0*.*102*	*0*.*095*	*0*.*024*	-	0.479	0.469	0.504	0.700	0.698	0.587	0.574	0.540	0.543	0.635	0.587	0.746	0.696	0.659	0.720	0.622	0.668	0.760	0.666	0.683	0.603	0.598	0.619	0.638	0.576	0.622	0.597	0.701	0.579
B1	*0*.*056*	*0*.*053*	*0*.*056*	*0*.*073*	-	0.358	0.485	0.639	0.630	0.618	0.580	0.583	0.519	0.603	0.611	0.694	0.629	0.641	0.737	0.575	0.598	0.755	0.629	0.654	0.533	0.572	0.566	0.654	0.550	0.565	0.545	0.655	0.605
B2	*0*.*036*	*0*.*059*	*0*.*034*	*0*.*055*	0.012	-	0.495	0.649	0.651	0.585	0.583	0.554	0.487	0.572	0.637	0.635	0.643	0.615	0.719	0.576	0.602	0.713	0.616	0.643	0.570	0.571	0.578	0.657	0.538	0.562	0.558	0.687	0.582
B3	*0*.*067*	*0*.*102*	*0*.*060*	*0*.*080*	*0*.*052*	*0*.*033*	-	0.737	0.738	0.597	0.533	0.533	0.574	0.642	0.622	0.705	0.665	0.683	0.745	0.671	0.697	0.748	0.619	0.641	0.617	0.636	0.635	0.654	0.586	0.635	0.623	0.708	0.643
C1	*0*.*230*	*0*.*218*	*0*.*233*	*0*.*211*	*0*.*174*	*0*.*154*	*0*.*233*	-	0.198	0.706	0.739	0.715	0.687	0.732	0.715	0.761	0.695	0.688	0.715	0.708	0.705	0.774	0.739	0.752	0.690	0.737	0.649	0.703	0.589	0.613	0.609	0.694	0.682
C2	*0*.*293*	*0*.*294*	*0*.*285*	*0*.*268*	*0*.*231*	*0*.*224*	*0*.*297*	0.006	-	0.714	0.746	0.721	0.685	0.749	0.725	0.762	0.681	0.676	0.731	0.713	0.703	0.801	0.736	0.761	0.698	0.744	0.667	0.698	0.593	0.614	0.627	0.682	0.701
D1	*0*.*215*	*0*.*229*	*0*.*178*	*0*.*182*	*0*.*184*	*0*.*139*	*0*.*178*	*0*.*326*	*0*.*374*	-	0.599	0.524	0.644	0.668	0.617	0.756	0.766	0.766	0.801	0.735	0.761	0.801	0.687	0.675	0.684	0.652	0.668	0.685	0.617	0.682	0.663	0.666	0.609
D2	*0*.*235*	*0*.*271*	*0*.*187*	*0*.*209*	*0*.*204*	*0*.*165*	*0*.*199*	*0*.*412*	*0*.*439*	*0*.*298*	-	0.313	0.645	0.660	0.660	0.720	0.658	0.682	0.780	0.691	0.734	0.732	0.642	0.637	0.645	0.638	0.640	0.708	0.575	0.646	0.647	0.731	0.627
D3	*0*.*187*	*0*.*223*	*0*.*136*	*0*.*164*	*0*.*172*	*0*.*132*	*0*.*149*	*0*.*364*	*0*.*408*	*0*.*241*	*0*.*041*	-	0.603	0.618	0.662	0.703	0.678	0.664	0.765	0.668	0.713	0.677	0.638	0.615	0.627	0.610	0.632	0.673	0.528	0.625	0.612	0.709	0.603
D4	*0*.*071*	*0*.*102*	*0*.*093*	*0*.*092*	*0*.*062*	*0*.*031*	*0*.*079*	*0*.*204*	*0*.*264*	*0*.*193*	*0*.*226*	*0*.*184*	-	0.502	0.649	0.673	0.679	0.647	0.727	0.632	0.681	0.698	0.646	0.704	0.594	0.648	0.593	0.618	0.574	0.585	0.579	0.731	0.602
D5	*0*.*098*	*0*.*128*	*0*.*127*	*0*.*130*	*0*.*084*	*0*.*050*	*0*.*111*	*0*.*264*	*0*.*339*	*0*.*251*	*0*.*272*	*0*.*216*	*0*.*028*	-	0.647	0.699	0.738	0.716	0.752	0.695	0.726	0.708	0.711	0.751	0.651	0.668	0.557	0.610	0.578	0.690	0.643	0.726	0.614
E1	*0*.*225*	*0*.*188*	*0*.*232*	*0*.*187*	*0*.*170*	*0*.*169*	*0*.*195*	*0*.*346*	*0*.*388*	*0*.*268*	*0*.*351*	*0*.*320*	*0*.*195*	*0*.*233*	-	0.725	0.695	0.709	0.678	0.690	0.680	0.794	0.642	0.641	0.560	0.565	0.666	0.651	0.644	0.637	0.583	0.647	0.541
E2	*0*.*183*	*0*.*249*	*0*.*240*	*0*.*250*	*0*.*206*	*0*.*164*	*0*.*227*	*0*.*320*	*0*.*373*	*0*.*345*	*0*.*339*	*0*.*299*	*0*.*187*	*0*.*215*	*0*.*336*	-	0.577	0.516	0.522	0.532	0.567	0.413	0.583	0.600	0.592	0.604	0.640	0.692	0.606	0.584	0.575	0.786	0.669
E3	*0*.*217*	*0*.*227*	*0*.*230*	*0*.*242*	*0*.*209*	*0*.*184*	*0*.*226*	*0*.*301*	*0*.*352*	*0*.*342*	*0*.*324*	*0*.*300*	*0*.*228*	*0*.*264*	*0*.*323*	*0*.*181*	-	0.283	0.478	0.540	0.507	0.612	0.457	0.573	0.474	0.492	0.661	0.693	0.568	0.503	0.528	0.613	0.665
E4	*0*.*198*	*0*.*208*	*0*.*203*	*0*.*218*	*0*.*196*	*0*.*162*	*0*.*209*	*0*.*274*	*0*.*328*	*0*.*322*	*0*.*310*	*0*.*282*	*0*.*198*	*0*.*234*	*0*.*310*	*0*.*144*	*0*.*026*	-	0.417	0.466	0.457	0.561	0.461	0.548	0.467	0.482	0.585	0.659	0.519	0.480	0.501	0.637	0.603
E5	*0*.*214*	*0*.*197*	*0*.*241*	*0*.*235*	*0*.*212*	*0*.*178*	*0*.*243*	*0*.*309*	*0*.*378*	*0*.*369*	*0*.*408*	*0*.*362*	*0*.*216*	*0*.*253*	*0*.*317*	*0*.*140*	*0*.*075*	*0*.*036*	-	0.538	0.519	0.575	0.523	0.647	0.554	0.559	0.635	0.713	0.621	0.581	0.616	0.672	0.648
E6	*0*.*126*	*0*.*156*	*0*.*149*	*0*.*154*	*0*.*121*	*0*.*104*	*0*.*151*	*0*.*240*	*0*.*300*	*0*.*268*	*0*.*288*	*0*.*252*	*0*.*126*	*0*.*164*	*0*.*254*	*0*.*114*	*0*.*124*	*0*.*088*	*0*.*096*	-	0.363	0.617	0.468	0.542	0.456	0.516	0.546	0.624	0.552	0.504	0.535	0.666	0.566
E7	*0*.*168*	*0*.*184*	*0*.*184*	*0*.*190*	*0*.*159*	*0*.*142*	*0*.*191*	*0*.*261*	*0*.*311*	*0*.*299*	*0*.*328*	*0*.*294*	*0*.*181*	*0*.*208*	*0*.*279*	*0*.*138*	*0*.*096*	*0*.*088*	*0*.*077*	*0*.*029*	-	0.622	0.454	0.564	0.439	0.510	0.595	0.666	0.568	0.512	0.553	0.634	0.558
E8	*0*.*272*	*0*.*329*	*0*.*349*	*0*.*333*	*0*.*300*	*0*.*256*	*0*.*324*	*0*.*434*	*0*.*476*	*0*.*434*	*0*.*436*	*0*.*380*	*0*.*280*	*0*.*334*	*0*.*430*	*0*.*143*	*0*.*254*	*0*.*222*	*0*.*254*	*0*.*227*	*0*.*241*	-	0.633	0.610	0.659	0.643	0.673	0.726	0.666	0.639	0.616	0.821	0.705
F1	*0*.*175*	*0*.*182*	*0*.*176*	*0*.*192*	*0*.*173*	*0*.*148*	*0*.*165*	*0*.*283*	*0*.*336*	*0*.*255*	*0*.*285*	*0*.*253*	*0*.*178*	*0*.*221*	*0*.*271*	*0*.*169*	*0*.*088*	*0*.*084*	*0*.*105*	*0*.*078*	*0*.*071*	*0*.*268*	-	0.403	0.423	0.460	0.587	0.605	0.519	0.506	0.532	0.640	0.578
F2	*0*.*155*	*0*.*181*	*0*.*155*	*0*.*177*	*0*.*136*	*0*.*110*	*0*.*139*	*0*.*298*	*0*.*370*	*0*.*249*	*0*.*272*	*0*.*225*	*0*.*160*	*0*.*209*	*0*.*277*	*0*.*152*	*0*.*142*	*0*.*101*	*0*.*178*	*0*.*090*	*0*.*116*	*0*.*238*	0.027	-	0.532	0.515	0.626	0.698	0.567	0.564	0.587	0.671	0.609
F3	*0*.*108*	*0*.*093*	*0*.*105*	*0*.*101*	*0*.*079*	*0*.*083*	*0*.*100*	*0*.*206*	*0*.*264*	*0*.*217*	*0*.*239*	*0*.*199*	*0*.*101*	*0*.*133*	*0*.*178*	*0*.*159*	*0*.*103*	*0*.*098*	*0*.*104*	*0*.*059*	*0*.*073*	*0*.*256*	*0*.*058*	*0*.*075*	-	0.366	0.554	0.563	0.449	0.428	0.445	0.628	0.591
F4	*0*.*122*	*0*.*124*	*0*.*103*	*0*.*103*	*0*.*104*	*0*.*094*	*0*.*124*	*0*.*227*	*0*.*286*	*0*.*214*	*0*.*236*	*0*.*195*	*0*.*131*	*0*.*156*	*0*.*194*	*0*.*169*	*0*.*099*	*0*.*098*	*0*.*104*	*0*.*078*	*0*.*084*	*0*.*230*	*0*.*073*	*0*.*070*	*0*.*036*	-	0.613	0.615	0.527	0.499	0.508	0.613	0.608
G1	*0*.*128*	*0*.*148*	*0*.*154*	*0*.*150*	*0*.*098*	*0*.*102*	*0*.*140*	*0*.*232*	*0*.*281*	*0*.*262*	*0*.*292*	*0*.*252*	*0*.*119*	*0*.*125*	*0*.*262*	*0*.*221*	*0*.*275*	*0*.*227*	*0*.*243*	*0*.*135*	*0*.*191*	*0*.*320*	*0*.*206*	*0*.*191*	*0*.*123*	*0*.*162*	-	0.479	0.451	0.509	0.499	0.655	0.537
G2	*0*.*234*	*0*.*234*	*0*.*250*	*0*.*213*	*0*.*196*	*0*.*190*	*0*.*223*	*0*.*321*	*0*.*350*	*0*.*337*	*0*.*397*	*0*.*350*	*0*.*184*	*0*.*216*	*0*.*313*	*0*.*319*	*0*.*329*	*0*.*298*	*0*.*336*	*0*.*229*	*0*.*275*	*0*.*411*	*0*.*263*	*0*.*285*	*0*.*179*	*0*.*207*	*0*.*173*	-	0.489	0.542	0.542	0.627	0.608
G3	*0*.*119*	*0*.*130*	*0*.*110*	*0*.*121*	*0*.*099*	*0*.*080*	*0*.*119*	*0*.*194*	*0*.*241*	*0*.*222*	*0*.*196*	*0*.*158*	*0*.*108*	*0*.*126*	*0*.*248*	*0*.*207*	*0*.*214*	*0*.*173*	*0*.*219*	*0*.*155*	*0*.*181*	*0*.*317*	*0*.*177*	*0*.*143*	*0*.*090*	*0*.*126*	*0*.*098*	*0*.*188*	-	0.391	0.429	0.620	0.528
G4	*0*.*095*	*0*.*122*	*0*.*108*	*0*.*109*	*0*.*081*	*0*.*067*	*0*.*105*	*0*.*163*	*0*.*215*	*0*.*217*	*0*.*222*	*0*.*191*	*0*.*081*	*0*.*126*	*0*.*212*	*0*.*141*	*0*.*148*	*0*.*113*	*0*.*128*	*0*.*075*	*0*.*095*	*0*.*229*	*0*.*110*	*0*.*092*	*0*.*050*	*0*.*062*	*0*.*076*	*0*.*154*	*0*.*054*	-	0.301	0.656	0.609
G5	*0*.*098*	*0*.*122*	*0*.*106*	*0*.*097*	*0*.*073*	*0*.*062*	*0*.*100*	*0*.*165*	*0*.*227*	*0*.*213*	*0*.*228*	*0*.*184*	*0*.*065*	*0*.*107*	*0*.*188*	*0*.*137*	*0*.*161*	*0*.*118*	*0*.*155*	*0*.*086*	*0*.*121*	*0*.*231*	*0*.*127*	*0*.*090*	*0*.*049*	*0*.*069*	*0*.*078*	*0*.*158*	*0*.*051*	-0.01	-	0.665	0.574
H1	*0*.*236*	*0*.*251*	*0*.*259*	*0*.*253*	*0*.*200*	*0*.*195*	*0*.*249*	*0*.*321*	*0*.*354*	*0*.*322*	*0*.*392*	*0*.*358*	*0*.*238*	*0*.*277*	*0*.*316*	*0*.*354*	*0*.*263*	*0*.*267*	*0*.*305*	*0*.*240*	*0*.*240*	*0*.*440*	*0*.*248*	*0*.*277*	*0*.*192*	*0*.*202*	*0*.*255*	*0*.*271*	*0*.*234*	*0*.*204*	*0*.*219*	-	0.578
H2	*0*.*144*	*0*.*188*	*0*.*194*	*0*.*163*	*0*.*135*	*0*.*113*	*0*.*167*	*0*.*281*	*0*.*339*	*0*.*253*	*0*.*318*	*0*.*279*	*0*.*132*	*0*.*167*	*0*.*207*	*0*.*260*	*0*.*283*	*0*.*234*	*0*.*264*	*0*.*172*	*0*.*204*	*0*.*355*	*0*.*232*	*0*.*218*	*0*.*165*	*0*.*195*	*0*.*148*	*0*.*276*	*0*.*156*	*0*.*144*	*0*.*126*	*0*.*245*	-

**Fig 2:**
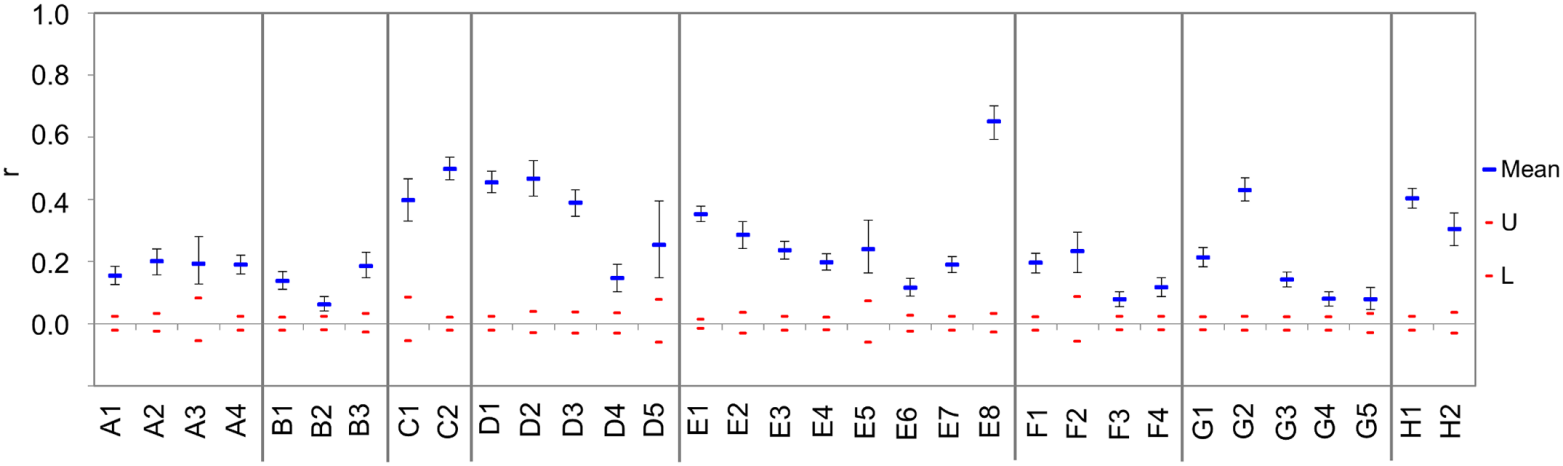
Correlogram representing the mean within-population pairwise similarities using the Lynch & Ritland (1999) estimator. Upper (U) and lower (L) confidence limits bound the 95% CI of the null hypothesis of 'No Difference' across the populations using 999 random permutations, and for estimates of r by bootstrapping 1,000 pairwise comparisons. Whiskers represent the highest and lowest values within a dataset for each distance class. Regions are separated by vertical grey lines. Codes of sites correspond to those of Table 1.

Mean within-population pairwise correlation relatedness values, “r”, ranged between 0.063 and 0.651, and were significant in all cases (Fig 2). These values correlated negatively with the observed heterozygosity and with Shannon's Information Index (Spearman’s rho: -0.7681, p<0.01, and -0.956, p<0.001 respectively). Therefore, higher “r” values refer to higher separation and lower genetic diversity (these features may indicate inbreeding). In some cases (e.g., populations G2 = 0.651, C2 = 0.498, D2 = 0.467), high values were found, but pronounced regional patterns were not. In almost all sampling regions (except regions A and B), one or more populations formed by strongly related individuals were observed.

**Fig 3:**
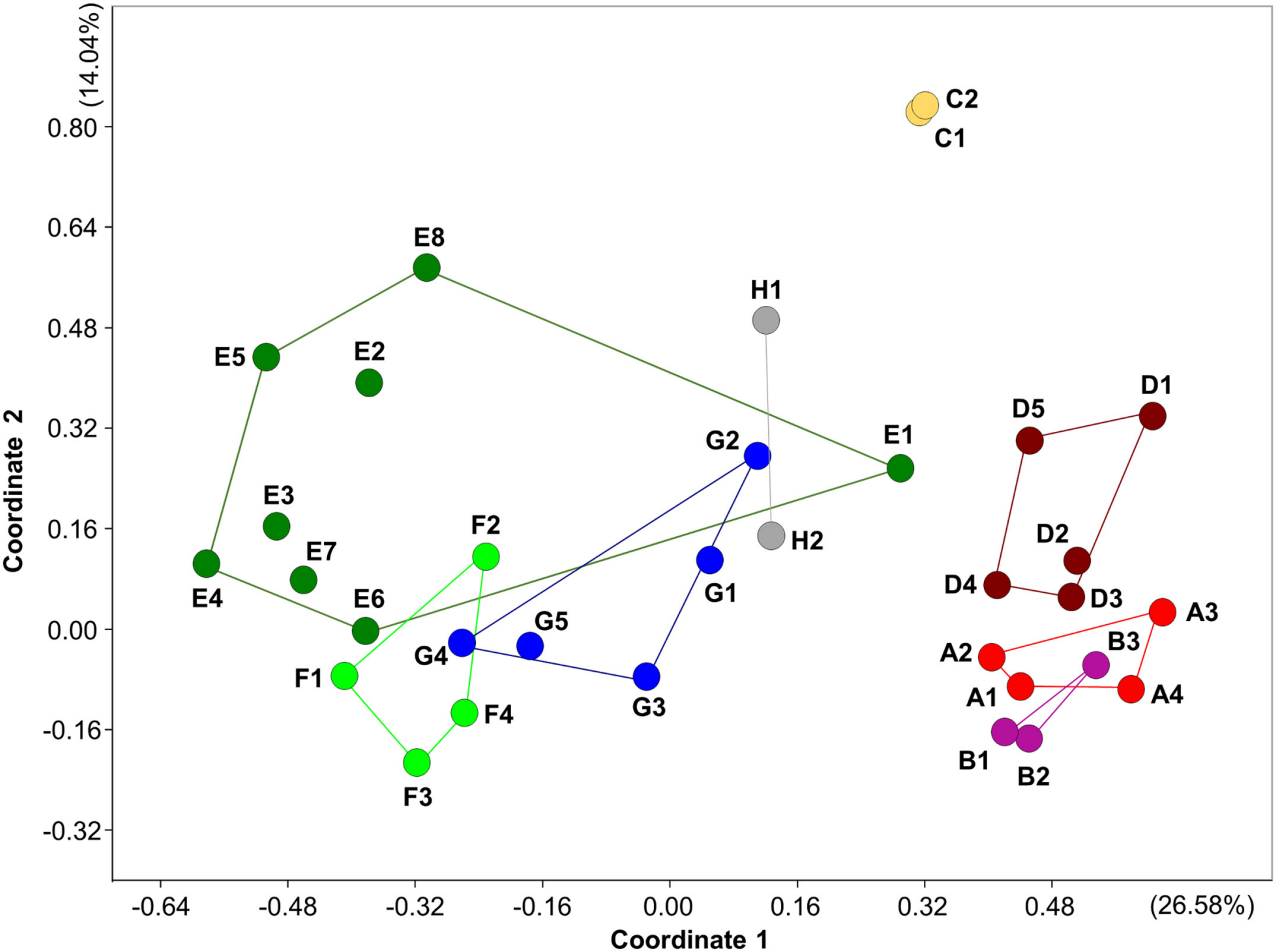
PCoA plot of pairwise population genetic distances (using Cavalli-Sforza and Edwards estimator after FREENA-INA null allele correction). Percentage variances represented by axes are shown in parentheses. Codes and colours of sites correspond to those of Table 1 and Fig 1.

The PCoA plot based on pairwise population genetic distances (Fig 3) showed clear separation along the “x” axis of eastern (Tisza drainage basin) and western (Danube drainage basin) sites. Moreover, the populations in the Tisza drainage basin showed higher similarity. In the case of these populations, the separation along the second axis is also important. Pronounced separation of neighbouring populations (i.e., populations from the same region) within a region can only be seen in the case of population E1. This population belongs to the Danube drainage basin Middle Hungarian region, but shows stronger similarity to the Mura region or to the C1, C2, and D4 populations of the Tisza drainage basin.

AMOVA analysis showed that among-population and among-regional differences represented 12.8% and 7.8% of the total variation respectively, while within-population differences accounted for 78.9% of the total genetic variance. Results of the regional F-statistics, except F_is_, showed significant within- and among-regional differentiation and population level differences.

**Fig 4:**
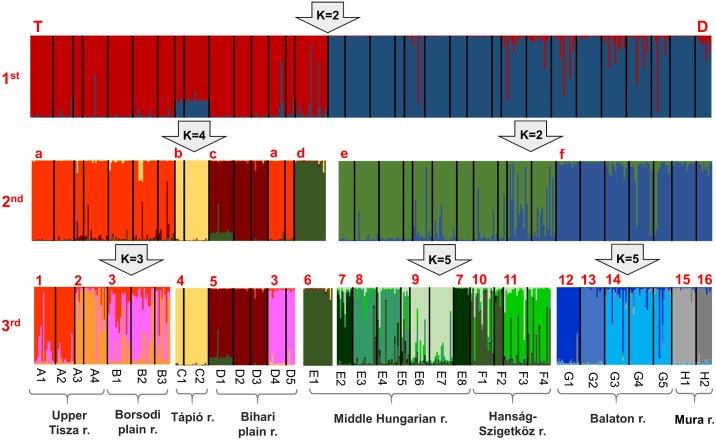
Estimated population structure as inferred by three rounds of hierarchical structure analyses. Each individual is represented by a thin horizontal line, which is partitioned into *K-*coloured segments representing individual’s estimated membership fractions in *K* *c*lusters. Black lines separate individuals from different sampling sites. The most probable K for a sample, given by the arrows, is based on the results of StructureHarvester using the Evanno method. Codes of sampling sites correspond to those of Table 1. Red codes represented on the three hierarchical levels correspond to the codes used in Fig 6.

Bayesian hierarchical clustering separated Danubian and Tiszaian mudminnow stocks in the first step (Fig 4). The only exception is the E1 population, which was assigned clearly to the Tiszanian clade. The Tiszanian group was split into 4 clusters; the E1 population, Tápió region (C1-2), and sites from NE Bihar region (D1-3) were separated from the others. Stocks form the Upper Tisza region (A1-4), Borsodi-mezőség region (B1-3), and SW of Bihar plain (D4-5) were separated only in the third clustering step, and the extent of separation is not as unambiguous as in the previous steps. The Danubian branch split to two larger groups; the Middle Hungarian region together with the Hanság-Szigetköz region (E+F) formed a group separated from the Balaton and Mura (G+H) regions. The E+F cluster split further into four minor groups (E2+E8; E3+E4+E5; E6+E7; F1-F4). Populations in the G+H cluster formed five minor groups. Distinct populations were found in this cluster, except G3-G4-G5 stocks, which all originated from the Kis-Balaton marshland area and formed one population.

**Fig 5:**
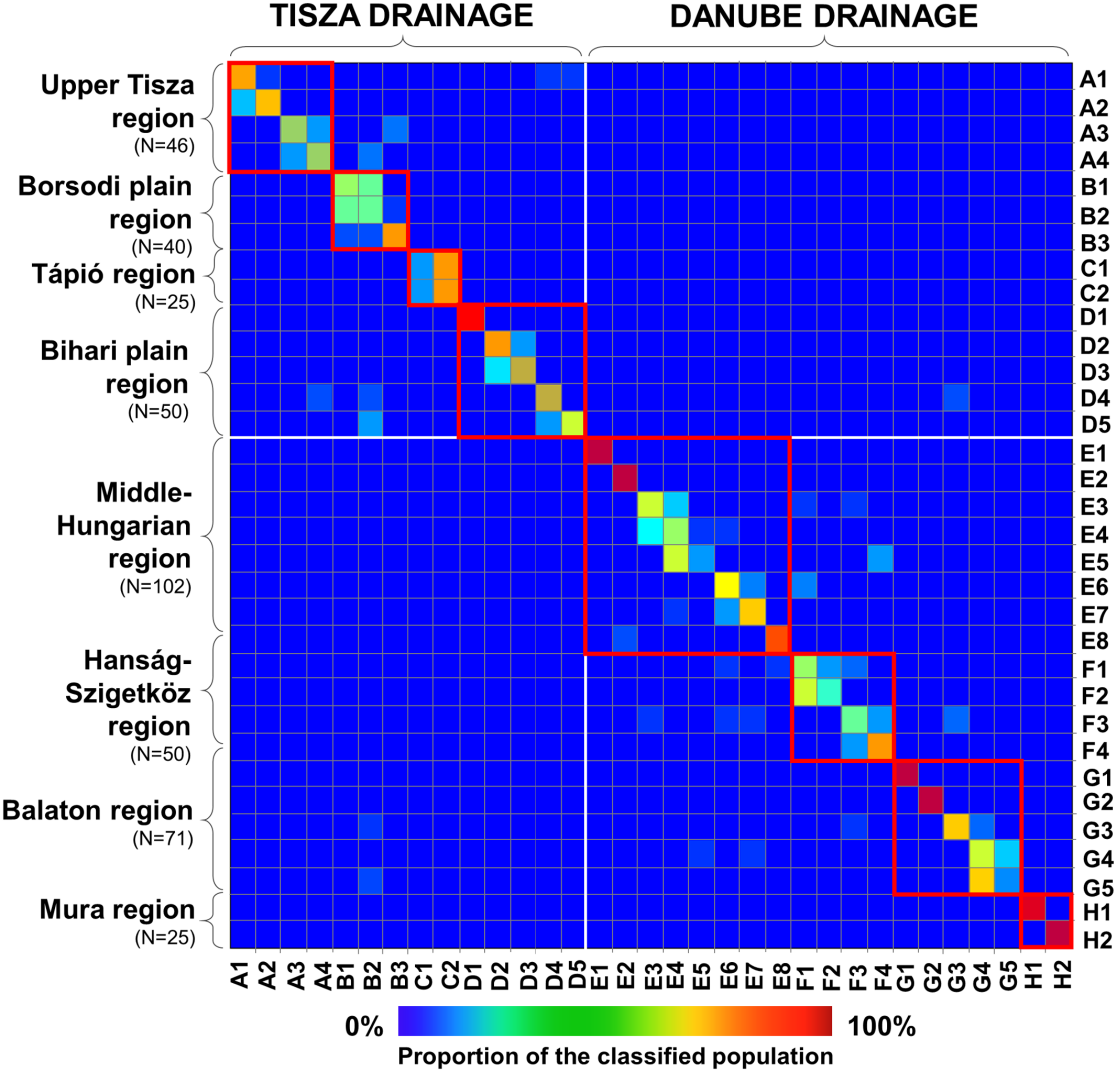
Plots of the Bayesian cross-validation test for microsatellite data. Correctly classified individuals are placed on the diagonal. The square colour corresponds to the proportion of individuals of posterior group assignment based on posterior probabilities. Rows correspond to actual sites (a priori), while columns correspond to inferred sites (posteriori). White thick lines separate the Tisza and Danube drainage basins, red rectangles show the regional detachments of populations. Codes of sites correspond to Table 1.

Bayesian cross-validation showed that 98.3%, 89.6%, and 71.8% of the individuals were grouped correctly on drainage basin, region and population levels respectively (Fig 5). Most of the reclassifications were made within the same region, mainly between neighbouring sites (e.g., C1-C2).

Result of the cross validation procedure showed negligible linkage between the Danubian and Tisza drainage basins. Only three specimens (i.e., 0.7% of the total sample) were misclassified between the two drainage basins. Within a drainage basin, the percentage of misclassified cases is higher; five specimens of the total 156 (i.e., 3.2%), and 13 specimens of the 248 (i.e., 5.2%) were classified to other regions on the Tisza and Danube drainage basins respectively. The highest number of misclassified cases was found between the Middle-Hungarian and Hanság-Szigetköz regions (E and F regions) where 4.9% and 10% of the stocks were misclassified.

Population assignment results from GeneClass2 strongly support the structure revealed on the three hierarchical levels identified by Bayesian STRUCTURE analyses. 97.8, 96.0 and 94.6% of the individuals were classified correctly on the three hierarchical levels respectively (see: Fig 4). The third level of the hierarchical STRUCTURE analyses was therefore found to have classified the individuals much more reliably than the original population based classification.

The between-region (A-H) migration ranged between 0.28 and 2.31 individuals per generation. Mutation-scaled immigration rate is less than one individual per generation in most cases, therefore negligible gene flow is assumed among the eight studied regions in the Carpathian Basin, especially in the case of regions C and H.

Migration computations between the two clusters (corresponding to the Danube and Tisza catchments, designated on the first level of hierarchical STRUCTURE analysis) showed that the Danube system received 8.19 (Θ = 2.4941 and M = 3.286) and released 7.24 (Θ = 2.5105 and M = 2.887) migrating individuals per generation to the Tisza drainage basin alone.

**Fig 6:**
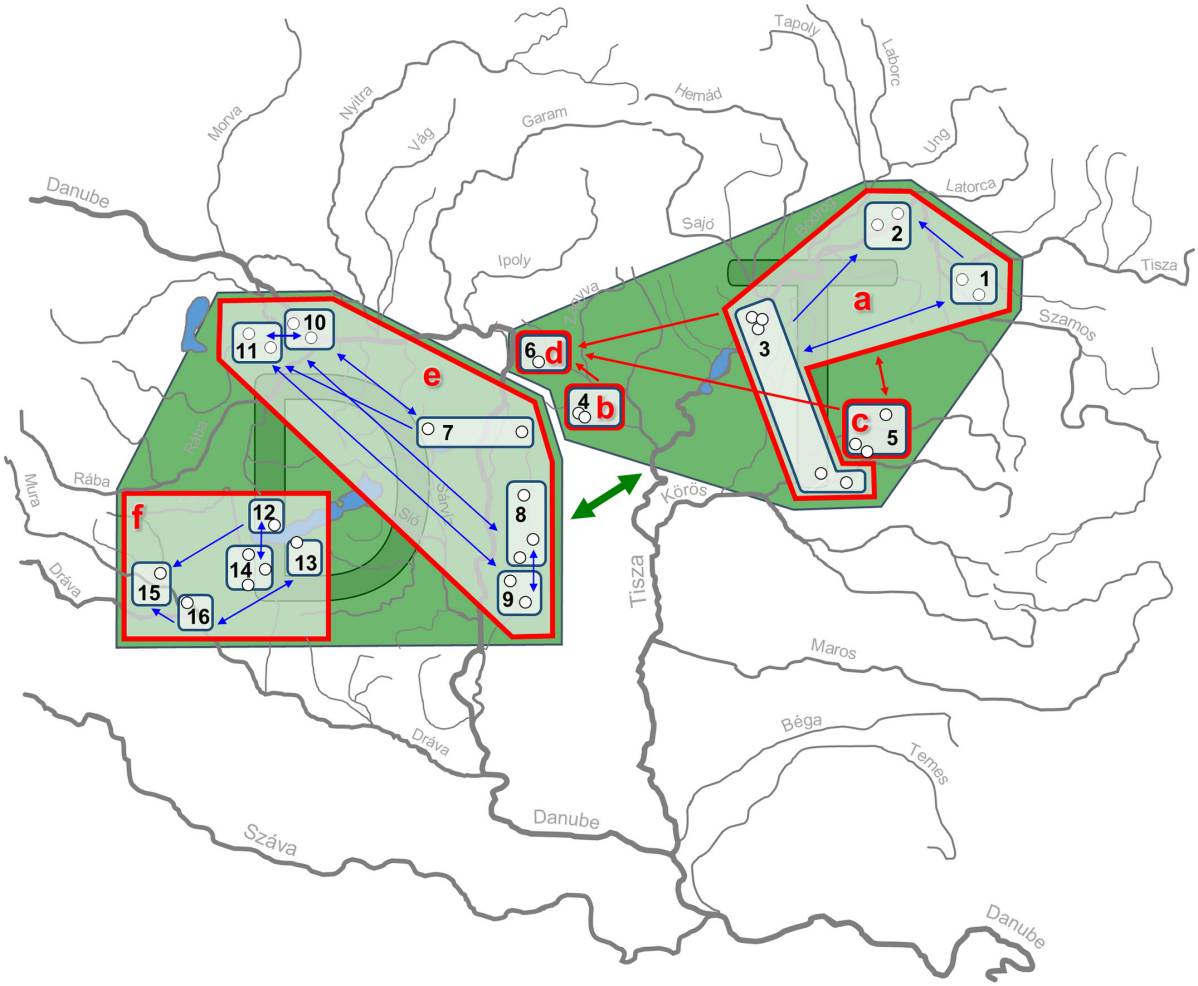
Migration within and among different clusters designated by hierarchical STRUCTURE analyses. Sites in the green areas belong to the same evolutionarily significant units (ESUs) identified by the 1^st^ level of hierarchical STRUCTURE analysis. Sites framed by red and blue lines belong to the same conservation units (CUs) and management units (MUs) designated by the 2^nd^ and 3^rd^ level by STRUCTURE analyses respectively. Arrows directions show between cluster migrations, where the migration rate is >1 individual per generation. Arrow colouring corresponds to hieararchical levels. Open circles show sampling sites. Codes of clusters correspond to Fig 4.

On the second level of STRUCTURE analysis, the immigration rate (M) between the four clusters (Figs 4 and 6) found in the Tisza catchment ranged between 0.75 and 1.52. The highest level of mutation-scaled immigration rate was found between the “a” and “c” clusters. In the case of the two Danubian clusters (see e and f clusters on Fig 4) the migration rates were less than one in both directions, resulting in a 1.36 and 1.48 individual per generation migration rate. These groups are therefore practically separated. Pairwise between-region and between-clusters immigration rates are presented in Tables 5–9.

**Table 5:** Migration computation among the eight hydrological regions preliminary defined, + receiving population (for region codes see Table 1).

	Θ	A+	B+	C+	D+	E+	F+	G+	H+
A	1.6931	-	1.048	0.620	1.186	1.075	0.886	0.901	0.802
B	1.7901	1.292	-	0.921	1.224	1.143	1.048	1.170	0.692
C	0.7948	0.858	0.967	-	0.713	1.128	1.238	0.750	0.964
D	1.2794	0.907	1.209	1.130	-	0.961	0.907	1.273	0.770
E	1.1628	1.199	1.124	1.224	0.909	-	1.055	1.602	0.847
F	1.3975	1.235	1.222	0.696	1.311	1.448	-	1.519	0.798
G	1.5376	1.211	0.830	1.161	1.060	1.200	1.053	-	0.923
H	0.4728	0.886	0.594	0.622	0.737	1.078	0.839	1.098	-

**Table 6:** Theta values (Θ) and immigration rates (M) of the six clusters defined in the second round of hierarchical STRUCTURE analysis (For cluster codes see Fig 4, + receiving population).

	Θ	a+	b+	c+	d+	e+	f+
a	2.0281	-	0.912	1.520	1.051	0.963	1.380
b	1.2179	0.911	-	0.804	1.161	0.758	1.061
c	1.3359	1.060	0.963	-	1.016	0.935	1.281
d	3.4662	0.926	0.749	0.720	-	0.866	0.802
e	1.7108	0.751	0.769	0.635	0.846	-	0.796
f	1.6411	1.726	0.977	1.071	0.861	0.902	-

**Table 7:** Within “a” cluster theta values (Θ) and immigration rates (M). Clusters defined in the third round of hierarchical STRUCTURE analysis. + receiving population (For cluster codes see Fig 4).

	Θ	1+	2+	3+
1	6.4478	-	1.014	1.030
2	9.8231	0.698	-	0.753
3	4.8238	1.519	1.091	-

**Table 8:** Within “e” cluster theta values (Θ) and immigration rates (M). Clusters defined in the third round of hierarchical STRUCTURE analysis. + receiving population (For cluster codes see Fig 4).

	Θ	7+	8+	9+	10+	11+
7	1.1948	-	0.846	1.055	0.781	1.475
8	2.4763	0.961	-	1.123	1.021	1.013
9	2.7797	0.685	1.132	-	1.055	1.552
10	1.8118	1.023	1.524	1.363	-	1.419
11	3.0720	0.529	1.120	1.270	0.942	-

**Table 9:** Within “f” cluster theta values (Θ) and immigration rates (M). Clusters defined in the third round of hierarchical STRUCTURE analysis. + receiving population (For cluster codes see Fig 4).

	Θ	12+	13+	14+	15+	16+
12	2.6243	-	0.772	1.153	1.113	0.757
13	1.7503	0.949	-	0.99	0.582	1.509
14	2.0176	1.293	0.992	-	0.782	0.799
15	0.6494	0.986	0.657	0.798	-	0.566
16	1.4528	0.921	1.196	0.831	1.000	-

**Fig 7:**
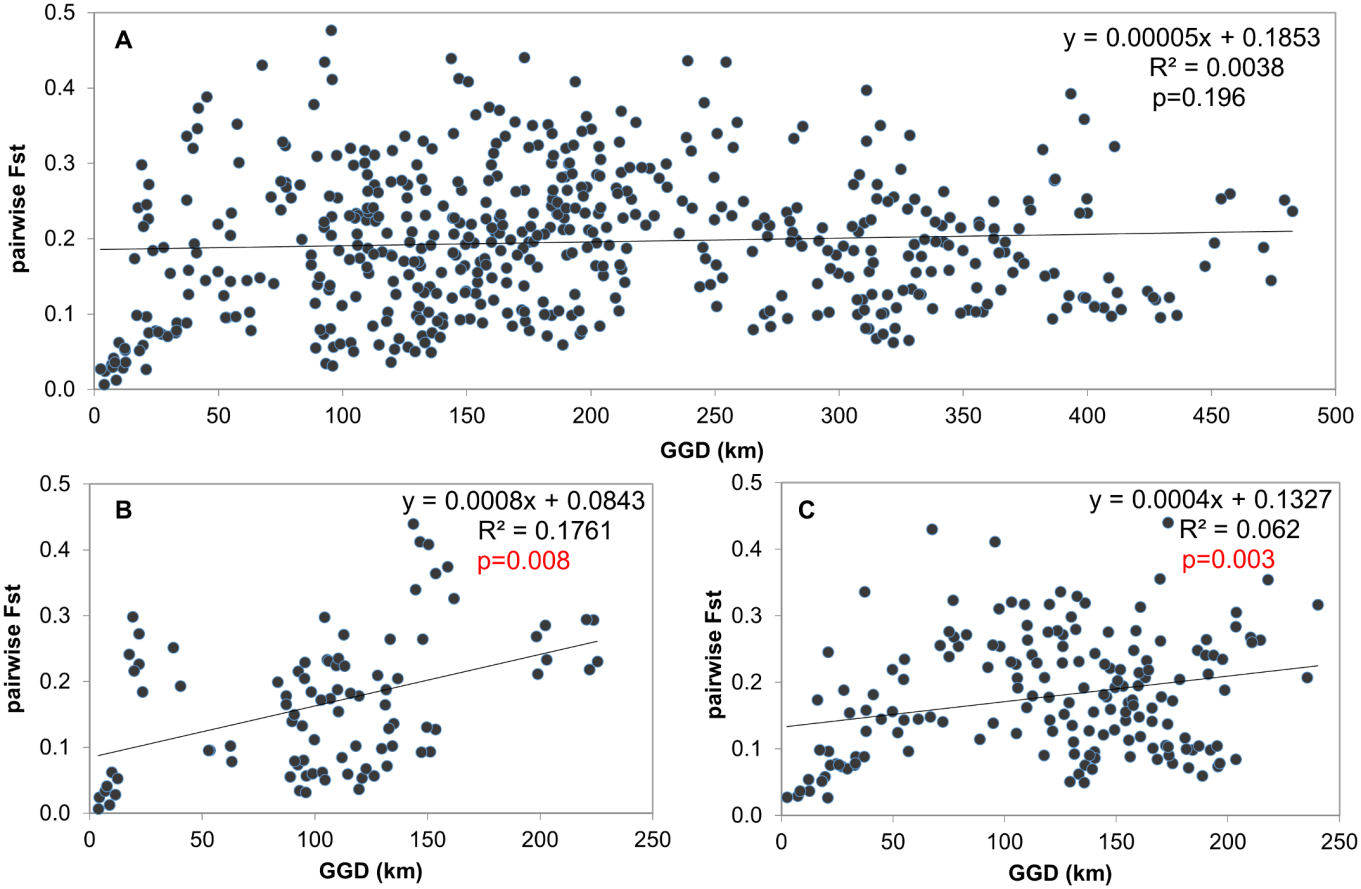
Results of Mantel tests conducted on the entire dataset (A), on the Tisza (B), and on the Danube drainage basin (C).

A Mantel test conducted on the entire dataset at the population level showed no significant correlation between pairwise *F*_*st*_ and GGD semimatrices (Rxy = 0.061, p>0.05). The inverse was found for the two drainage basins, where the genetic differences showed significant correlations both for the Tisza (Rxy = 0.4196, p<0.01) and the Danubian drainage basins (Rxy = 0.2489, p<0.01) as well. Therefore, population level IBD can be detected only within the Danubian and Tisza drainage basins (see Fig 7)

**Fig 8:**
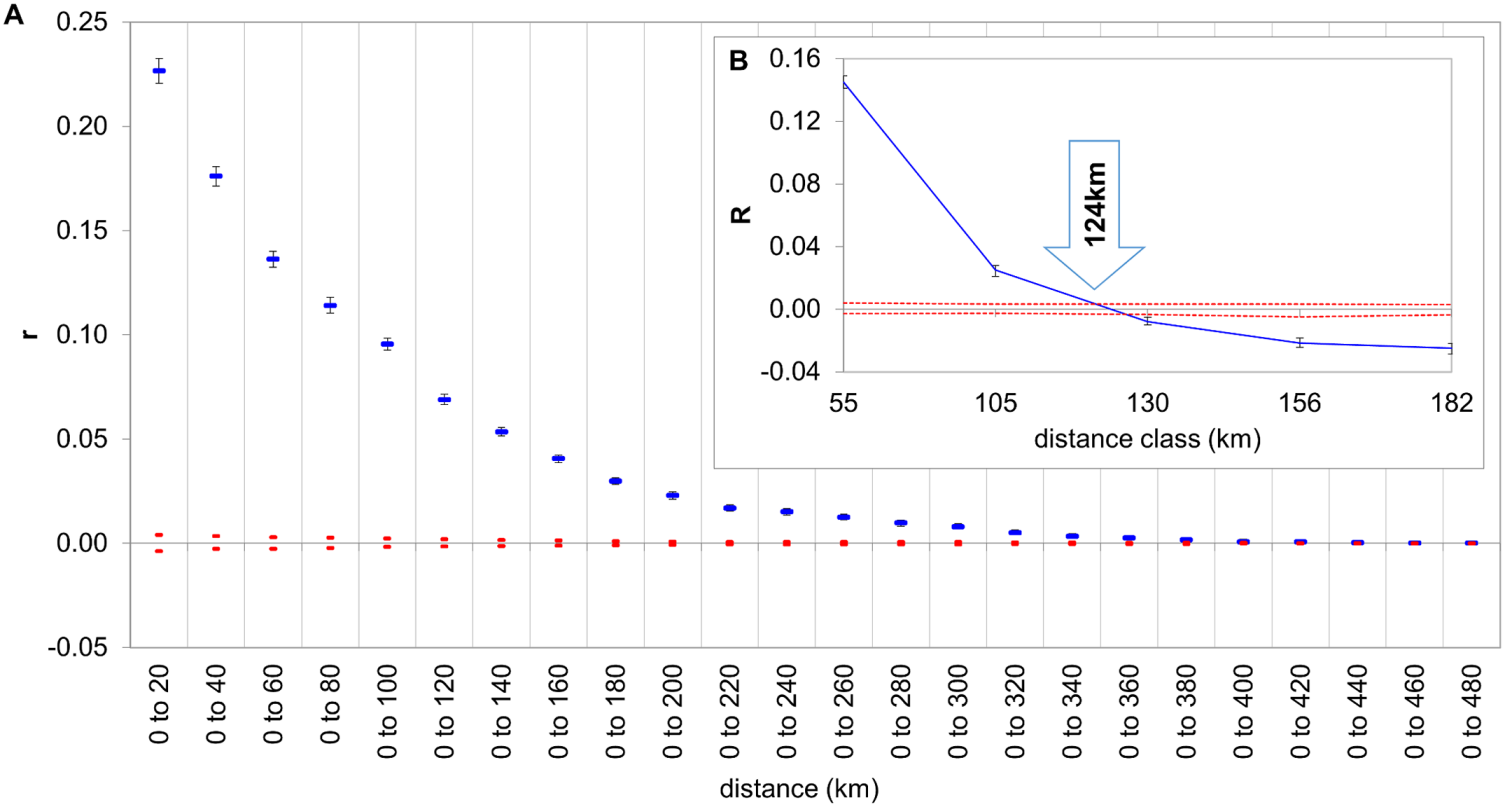
Correlograms showing autocorrelations calculated for the entire dataset. Coefficient “r” as a function of increasing cumulated distances (A). Correlogram “B” showing “R” as a function of distance classes designated by a similar number of pairwise comparisons for each class. Intercept value for the “x” axis is 124 km. Red dotted lines show the upper and lower confidence limits bounding the 95% CI of the null hypothesis of 'No Difference' across the populations using 999 random permutations, and for estimates of correlation coefficients by bootstrapping 1,000 pairwise comparisons for each distance class. Whiskers represent the highest and lowest values within a dataset.

**Fig 9:**
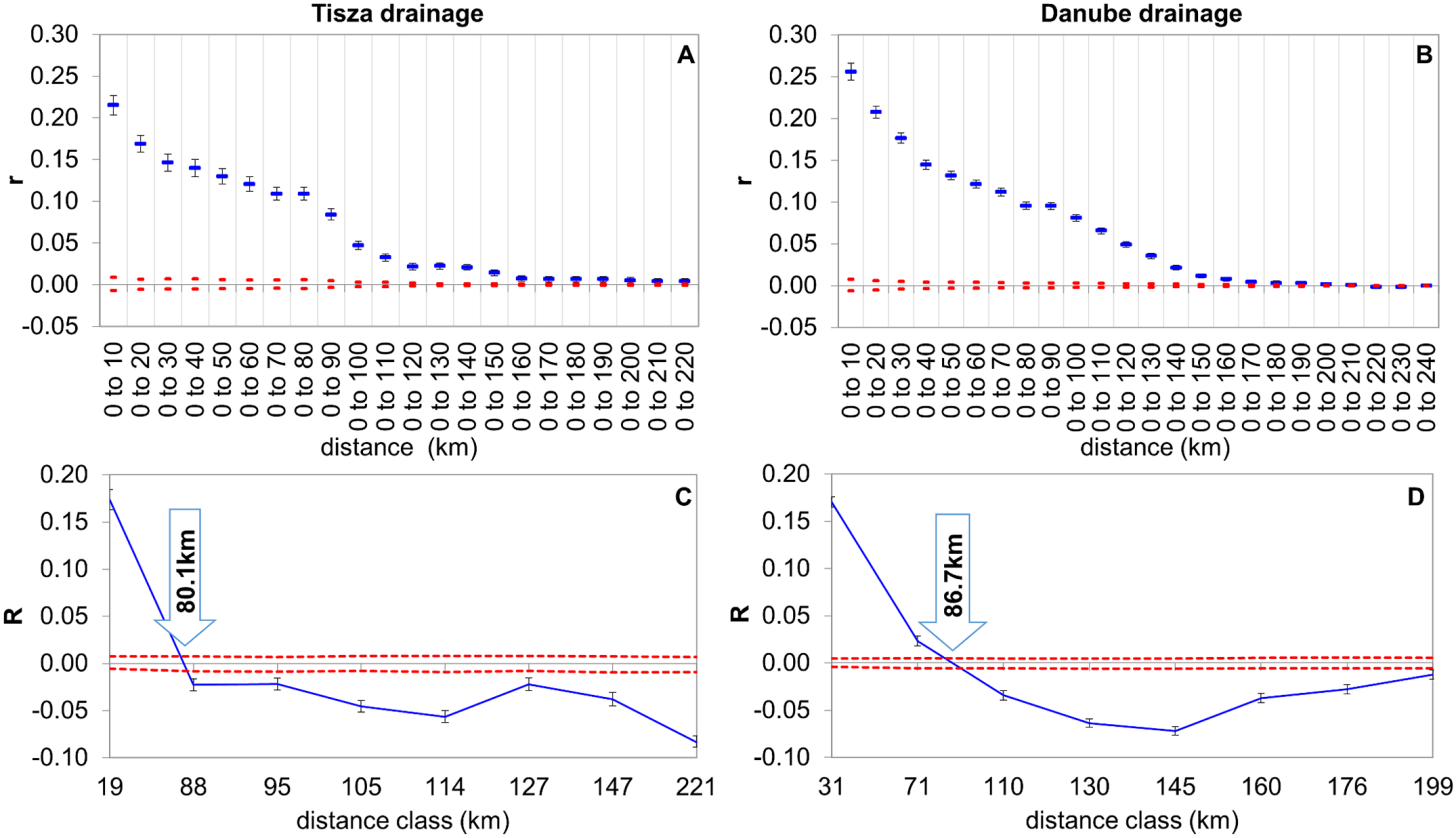
Correlograms showing the autocorrelation coefficients as a function of increasing distances (“*r*”) and distance classes (“R”) designated by a similar number of pairwise comparisons for each class, for the Tisza (A, C) and Danube drainage basin (B, D) respectively. Red dotted lines show the upper and lower confidence limits bounding the 95% CI of the null hypothesis of 'No Difference' across the populations using 999 random permutations, and for estimates of r by bootstrapping 1,000 pairwise comparisons for each distance class. Whiskers represent the highest and lowest values within a dataset. Intercept values for the “x” axes are 80.1 and 86.7kms for the Tisza and Danube drainage basins respectively.

Spatial autocorrelation computation carried out on the entire dataset at the individual level showed that IBD was not appreciable beyond an approximate 340–360 km distance. There was a significant positive correlation for the first two distance classes, which intercepted the “x” axes at 124 km (Fig 8A and 8B) and was therefore significant within this range. Autocorrelation calculation at the drainage basin level showed similar results in both cases. The IBD is still detectable to approximately 150–160 km, but it is significant only within an 80.1 and 86.7 km range for the Tisza and Danube drainage basins respectively (see Fig 9).

## Discussion

Tockner et al. [4] noted that fragmentation, water stress, land use change, and the introduction of non-native species are the most important threats to freshwater biodiversity. All these factors currently threaten mudminnow stocks, and their impacts are reflected in present day distribution and population genetic patterns. In this study, population genetic structure and dynamics of genetic mixing among populations in a strongly altered and fragmented landscape were analysed, and revealed some basic principles of optimal species conservation strategies.

### Distribution patterns

The field surveys of the current work have provided new information about the present day distribution of the European mudminnow at the centre of its range. In the “C” and “F” regions, stable and large assemblages were found, contrary to earlier communication which noted that the mudminnow was extremely rare and had been extirpated from most habitats in these regions [32]. At the same time, due to the expansion of Amur sleeper (*Perccottus glenii*) [70], European mudminnow has disappeared from many locations in the Hungarian Upper Tisza region (i.e., region “A” of this study), where it had previously been abundant [44]. At present, these shrinking populations seem to be the most threatened ones within the Carpathian Basin.

In almost all regions, the largest assemblages were found in artificial habitats. Of the 33 sampling sites, only seven were in a near-pristine state (Table 1). Consequently, similar to other threatened stagnophilous fish, it seems that some of the human-altered or man-made habitats (e.g., irrigation and drainage canals) with rich macrophyte coverage and permanent water supply might be the last chance to provide refuge for the shrinking European mudminnow populations [27, 71].

### Genetic and spatial structure

This study presents the first data on the population genetic structure of threatened and endemic European mudminnow for the centre of its distribution range. In general, remarkable genetic differentiation was found among the 33 populations originating from eight regions. The spatial structure revealed seems to be quite fixed with negligible migration among the studied regions (Table 5). The genetic structure corresponded clearly to past and recent geographical migratory routes (i.e., it reflected natural distribution patterns). The only identified anthropogenic alteration within this pattern is a result of a documented stock transfer from Middle Hungary to the Hanság-Szigetköz region (i.e., from region E to region F) in April 2005 [42]. Results of Bayesian cross validation tests (Fig 5), confirmed by migration tests (Fig 6), suggest that 10% and 5% of the specimens occurring here are closely related to Middle Hungarian (E) and Hanság-Szigetköz (F) populations respectively (Figs 5 and 6; Tables 5–9.). An alternative explanation for the mixed genetic composition of the Hanság-Szigetköz (F) region population could be the dispersion along the Danube valley route, as has been proven for many other species [72, 73]. In any case, SRUCTURE analysis showed (Fig 4) that the aforementioned stocking activity had only a moderate effect on the genetic pattern of mudminnow assemblages inhabiting this region.

Some of the studied populations showed complete isolation and a high level of relatedness (Figs 2 and 5). Populations which are characterised by special environmental requirements and restricted expansion ability may hold unique pieces of genetic diversity, and their loss can therefore cause a significant reduction in genetic diversity in general [74]. Therefore, to conserve the genetic diversity of this species, special attention must be paid to the preservation of these separated stocks (e.g., B2, D1, and G2 on Fig 2).

Although European mudminnow has a restricted dispersal ability [28], and historical processes therefore mainly underlie the pronounced spatial pattern in the genetic structure, our analyses also captured some human-induced effects related to the anthropogenic modification of the Carpathian Basin floodplains. Historical hydrological data [75, 76] suggest that prior to river regulation there were no notable barriers among some sub-drainage basins in the floodplain of the Tisza River (Fig 1). Periodic floods inundated almost the entire floodplain, sometimes for more than 3 months at a time (see: [22]), and migration could therefore take place freely among A-B-D regions in the Tisza drainage basin. As a result of river regulation, which began in 1846 and ended in the 1930s [77], the connections between regions became much more restricted. The strong distinctiveness of populations inhabiting the Tápió region (i.e., the “C” region here), outside the main floodplain, also supports this hypothesis (Fig 3).

According to data from the literature (cited in: [28, 39]), mudminnow may spawn at the age of one year for the first time. The separation of some Tiszanian regions could therefore have begun more than 100 generations ago. The separation of the Danubian and Tiszanian systems, as well as the mudminnow stocks, is presumably much older. The within-region separations are not complete in many cases, however, because the continuous flood-protection and regulation activity (i.e., dredging, flow direction shifts within the drain-canal systems) could have caused the connections between stocks to be re-established from time to time [78]. E1 is the only population among the 33 studied which seems to be misclassified by both the STRUCTURE and the pairwise population genetic distance analyses (Figs 3 and 4). Although this site currently belongs to the Danubian drainage system, the E1 population seems to be more related to those of the Tisza catchment. This relationship can be explained by paleohydrological changes caused by the emergence of Gödöllő Hilly Region in the Quaternary period [79], when the river system of this area was partly disjointed and the flow direction of the upper sections of the streams were diverted to the NW (i.e., to the Danube). At the same time, the two catchment areas were separated only by marshy areas referred to as “intra-valley drainage basin divides” [80], which these assemblages can traverse. This assumption is supported by the fact that the closest populations (C1-C2) to the Tápió system showed the highest similarities and bidirectional gene flow was found between the two areas. However, it should be noted that both assemblages showed high levels of isolation and inbreeding, and the connection between them is therefore presumably strictly limited. Similarly, contact between the G and H regions is plausible, because the isolation of the Middle and South Danubian catchments started only at the end of Pleistocene (by the formulation of the Lake Balaton basin) and this separation is still not complete [81]. Our results correspond with the findings of Brauer et al. [11], who investigated a South Australian endemic fish species with very similar environmental requirements to those of European mudminnow, the pygmy perch (*Nannoperca obscura* Klunzinger, 1872), and found similarly high levels of isolation, and designed CUs in very similar ranges. These results therefore show that preferred habitat type and dispersion abilities may have major roles in the formulation of these features rather than the geographic range (i.e., Australia vs. Europe) or the taxonomic position (i.e., Perciformes vs. Esociformes) of the studied species.

### Implications for conservation and management

Although pairwise *F*_*st*_ computation showed significant differentiation in most cases, none of the small stocks differed considerably from their neighbouring populations (Table 5; Fig 5). Moreover, these small stocks did not carry any private alleles (Table 3). It is therefore likely that these small stocks belonged to a larger metapopulation system, and could have been isolated only recently. Therefore, special consideration for their preservation is necessary only if there is no larger population nearby presenting the same genetic features.

Based on the current data, it is recommended that any reconstructed or newly established habitats should be stocked by individuals originating from the same CU, namely from populations within an 80–90 km range. The 16 clusters separated in the third step of the hierarchical STRUCTURE analysis can be accepted as MUs for conservation, instead of populations. In many cases, the MUs agree with some strongly isolated populations (e.g., populations C1, E1, G1, and G2). These separate entities need special attention to prevent substantial genetic diversity loss. The *ex situ* preservation of these populations could also be considered, preferably by artificially recruiting and translocating them to fish-free revitalized or newly established nature-like habitats (see e.g., [43]).

## Conclusions

The investigations reported here reveal unexpectedly high genetic diversity of this endemic fish species, despite its declining and fragmented populations. The studied stocks show high levels of genetic variability, and this pattern seems to have been only slightly influenced by anthropogenic impacts (i.e., resettlements) so far. Continued habitat loss, along with the invasion of non-native competitors, strongly threaten this habitat specialist, endemic fish. Climate change increases the probability and severity of dry periods in the area, and represents a high risk for European mudminnow with its shrunken and fragmented population structure [82]. Consequently, there is a strong need for implementing comprehensive conservation management programs. Nevertheless, the extremely small and/or isolated populations (e.g., in region “C”) would require special attention, and thus should be prioritized for conservation.

## Supporting Information

S1 Table Summary of Chi-Square Tests for Hardy-Weinberg Equilibrium. (DOCX)

S2 Table Microsatellite data of Carpathian Umbra stocks (404 individuals, 33 populations, 8 regions). (DOCX)

S3 Table Results of null allele analyses using MICROCHECKER software. (DOCX)
