# Supplementary material for: Population Genetic Patterns of Threatened European Mudminnow (Umbra krameri Walbaum, 1792) in a Fragmented Landscape: Implications for Conservation Management
Source: PLoS One. 2015 Sep 22;10(9):e0138640. doi: 10.1371/journal.pone.0138640 (PMC4578892; doi:10.1371/journal.pone.0138640)
Supplement: S1 Table — (DOCX) [file pone.0138640.s001.docx]

| S1 Table |  |  |  |  |  |
| --- | --- | --- | --- | --- | --- |
| Summary of Chi-Square Tests for Hardy-Weinberg Equilibrium | | | | | |
|  |  |  |  |  |  |
| No. Loci | 8 |  |  |  |  |
| No. Samples | 404 |  |  |  |  |
| No. Pops. | 33 |  |  |  |  |
|  |  |  |  |  |  |
| Pop | Locus | DF | ChiSq | Prob | Signif |
| A1 | UkrTet1 | 21 | 22.508 | 0.371 | ns |
| A1 | UkrTet3 | 78 | 63 | 0.891 | ns |
| A1 | UkrTet4 | 45 | 33.056 | 0.907 | ns |
| A1 | UkrTet5 | 10 | 7.055 | 0.72 | ns |
| A1 | UkrTet6 | 1 | 0.032 | 0.859 | ns |
| A1 | UkrTet7 | 28 | 25.076 | 0.624 | ns |
| A1 | UkrTet8 | 15 | 33.668 | 0.004 | ** |
| A1 | UkrTet9 | 21 | 34.362 | 0.033 | * |
| A2 | UkrTet1 | 28 | 19.917 | 0.867 | ns |
| A2 | UkrTet3 | 66 | 57.667 | 0.758 | ns |
| A2 | UkrTet4 | 10 | 4.859 | 0.9 | ns |
| A2 | UkrTet5 | 10 | 9.37 | 0.497 | ns |
| A2 | UkrTet6 | 1 | 0.023 | 0.88 | ns |
| A2 | UkrTet7 | 10 | 3.846 | 0.954 | ns |
| A2 | UkrTet8 | 21 | 23.103 | 0.338 | ns |
| A2 | UkrTet9 | 15 | 21.248 | 0.129 | ns |
| A3 | UkrTet1 | 15 | 15 | 0.451 | ns |
| A3 | UkrTet3 | 10 | 6.667 | 0.756 | ns |
| A3 | UkrTet4 | 3 | 0.625 | 0.891 | ns |
| A3 | UkrTet5 | 6 | 2.222 | 0.898 | ns |
| A3 | UkrTet6 | 1 | 0.313 | 0.576 | ns |
| A3 | UkrTet7 | 6 | 6.25 | 0.396 | ns |
| A3 | UkrTet8 | 6 | 8.75 | 0.188 | ns |
| A3 | UkrTet9 | 28 | 27.5 | 0.491 | ns |
| A4 | UkrTet1 | 45 | 40.698 | 0.655 | ns |
| A4 | UkrTet3 | 36 | 32.778 | 0.623 | ns |
| A4 | UkrTet4 | 10 | 30.441 | 0.001 | *** |
| A4 | UkrTet5 | 10 | 11.716 | 0.305 | ns |
| A4 | UkrTet6 | 1 | 0.355 | 0.551 | ns |
| A4 | UkrTet7 | 10 | 12.958 | 0.226 | ns |
| A4 | UkrTet8 | 21 | 17.364 | 0.689 | ns |
| A4 | UkrTet9 | 78 | 83.519 | 0.314 | ns |
| B1 | UkrTet1 | 55 | 52.545 | 0.569 | ns |
| B1 | UkrTet3 | 55 | 54.619 | 0.489 | ns |
| B1 | UkrTet4 | 45 | 36.724 | 0.805 | ns |
| B1 | UkrTet5 | 36 | 26.165 | 0.886 | ns |
| B1 | UkrTet6 | 1 | 0.077 | 0.782 | ns |
| B1 | UkrTet7 | 36 | 36.192 | 0.46 | ns |
| B1 | UkrTet8 | 45 | 39.65 | 0.697 | ns |
| B1 | UkrTet9 | 45 | 38.458 | 0.744 | ns |
| B2 | UkrTet1 | 78 | 69.583 | 0.741 | ns |
| B2 | UkrTet3 | 105 | 122.917 | 0.112 | ns |
| B2 | UkrTet4 | 45 | 56.261 | 0.121 | ns |
| B2 | UkrTet5 | 36 | 34.65 | 0.533 | ns |
| B2 | UkrTet6 | 1 | 5.947 | 0.015 | * |
| B2 | UkrTet7 | 36 | 29.25 | 0.78 | ns |
| B2 | UkrTet8 | 45 | 52.375 | 0.21 | ns |
| B2 | UkrTet9 | 66 | 54.529 | 0.842 | ns |
| B3 | UkrTet1 | 36 | 35.378 | 0.498 | ns |
| B3 | UkrTet3 | 36 | 43.333 | 0.187 | ns |
| B3 | UkrTet4 | 15 | 9.881 | 0.827 | ns |
| B3 | UkrTet5 | 3 | 2.104 | 0.551 | ns |
| B3 | UkrTet6 | 1 | 0.123 | 0.725 | ns |
| B3 | UkrTet7 | 21 | 15.079 | 0.819 | ns |
| B3 | UkrTet8 | 15 | 24.267 | 0.061 | ns |
| B3 | UkrTet9 | 10 | 6.494 | 0.772 | ns |
| C1 | UkrTet1 | 1 | 0.062 | 0.804 | ns |
| C1 | UkrTet3 | 15 | 11.25 | 0.735 | ns |
| C1 | UkrTet4 | 3 | 3.75 | 0.29 | ns |
| C1 | UkrTet5 | 3 | 3.75 | 0.29 | ns |
| C1 | UkrTet6 | Monomorphic | |  |  |
| C1 | UkrTet7 | 3 | 2.689 | 0.442 | ns |
| C1 | UkrTet8 | 3 | 0.918 | 0.821 | ns |
| C1 | UkrTet9 | 3 | 1.133 | 0.769 | ns |
| C2 | UkrTet1 | 6 | 10.207 | 0.116 | ns |
| C2 | UkrTet3 | 21 | 11.692 | 0.948 | ns |
| C2 | UkrTet4 | 3 | 2.103 | 0.551 | ns |
| C2 | UkrTet5 | 3 | 0.26 | 0.967 | ns |
| C2 | UkrTet6 | Monomorphic | |  |  |
| C2 | UkrTet7 | 6 | 2.63 | 0.854 | ns |
| C2 | UkrTet8 | 1 | 0.185 | 0.667 | ns |
| C2 | UkrTet9 | 3 | 2.083 | 0.555 | ns |
| D1 | UkrTet1 | 1 | 0.185 | 0.667 | ns |
| D1 | UkrTet3 | 3 | 2.018 | 0.569 | ns |
| D1 | UkrTet4 | 1 | 0.579 | 0.447 | ns |
| D1 | UkrTet5 | 10 | 8.219 | 0.607 | ns |
| D1 | UkrTet6 | Monomorphic | |  |  |
| D1 | UkrTet7 | 3 | 4.167 | 0.244 | ns |
| D1 | UkrTet8 | 10 | 8.719 | 0.559 | ns |
| D1 | UkrTet9 | 3 | 17.623 | 0.001 | *** |
| D2 | UkrTet1 | 10 | 16.511 | 0.086 | ns |
| D2 | UkrTet3 | 6 | 9.733 | 0.136 | ns |
| D2 | UkrTet4 | 6 | 4.249 | 0.643 | ns |
| D2 | UkrTet5 | 6 | 9.531 | 0.146 | ns |
| D2 | UkrTet6 | 1 | 0.028 | 0.868 | ns |
| D2 | UkrTet7 | 3 | 0.311 | 0.958 | ns |
| D2 | UkrTet8 | 3 | 4.067 | 0.254 | ns |
| D2 | UkrTet9 | 6 | 2.6 | 0.857 | ns |
| D3 | UkrTet1 | 15 | 7.686 | 0.936 | ns |
| D3 | UkrTet3 | 6 | 4.8 | 0.57 | ns |
| D3 | UkrTet4 | 10 | 12.158 | 0.275 | ns |
| D3 | UkrTet5 | 6 | 20.156 | 0.003 | ** |
| D3 | UkrTet6 | Monomorphic | |  |  |
| D3 | UkrTet7 | 6 | 0.625 | 0.996 | ns |
| D3 | UkrTet8 | 6 | 3.75 | 0.71 | ns |
| D3 | UkrTet9 | 15 | 13.6 | 0.556 | ns |
| D4 | UkrTet1 | 36 | 61.8 | 0.005 | ** |
| D4 | UkrTet3 | 28 | 26.667 | 0.536 | ns |
| D4 | UkrTet4 | 28 | 20.833 | 0.832 | ns |
| D4 | UkrTet5 | 21 | 20.816 | 0.47 | ns |
| D4 | UkrTet6 | 1 | 0.028 | 0.868 | ns |
| D4 | UkrTet7 | 36 | 30.556 | 0.725 | ns |
| D4 | UkrTet8 | 21 | 16.25 | 0.755 | ns |
| D4 | UkrTet9 | 28 | 35.378 | 0.159 | ns |
| D5 | UkrTet1 | 36 | 40 | 0.297 | ns |
| D5 | UkrTet3 | 10 | 8.75 | 0.556 | ns |
| D5 | UkrTet4 | 6 | 6.25 | 0.396 | ns |
| D5 | UkrTet5 | 6 | 5.756 | 0.451 | ns |
| D5 | UkrTet6 | Monomorphic | |  |  |
| D5 | UkrTet7 | 6 | 10.2 | 0.116 | ns |
| D5 | UkrTet8 | 6 | 5.556 | 0.475 | ns |
| D5 | UkrTet9 | 10 | 8.75 | 0.556 | ns |
| E1 | UkrTet1 | 15 | 28.465 | 0.019 | * |
| E1 | UkrTet3 | 55 | 59.433 | 0.317 | ns |
| E1 | UkrTet4 | 6 | 2.413 | 0.878 | ns |
| E1 | UkrTet5 | 1 | 0.078 | 0.78 | ns |
| E1 | UkrTet6 | 1 | 0.013 | 0.909 | ns |
| E1 | UkrTet7 | 6 | 1.792 | 0.938 | ns |
| E1 | UkrTet8 | 6 | 7.911 | 0.245 | ns |
| E1 | UkrTet9 | 15 | 17.297 | 0.301 | ns |
| E2 | UkrTet1 | 15 | 12.5 | 0.641 | ns |
| E2 | UkrTet3 | 55 | 58.611 | 0.344 | ns |
| E2 | UkrTet4 | 10 | 12.511 | 0.252 | ns |
| E2 | UkrTet5 | 1 | 0.023 | 0.88 | ns |
| E2 | UkrTet6 | 6 | 8.125 | 0.229 | ns |
| E2 | UkrTet7 | 10 | 6.927 | 0.732 | ns |
| E2 | UkrTet8 | 3 | 1.837 | 0.607 | ns |
| E2 | UkrTet9 | 6 | 4.6 | 0.596 | ns |
| E3 | UkrTet1 | 36 | 70.25 | 0.001 | *** |
| E3 | UkrTet3 | 45 | 48.958 | 0.317 | ns |
| E3 | UkrTet4 | 21 | 10.781 | 0.967 | ns |
| E3 | UkrTet5 | 6 | 12.507 | 0.052 | ns |
| E3 | UkrTet6 | 3 | 0.938 | 0.816 | ns |
| E3 | UkrTet7 | 10 | 8.97 | 0.535 | ns |
| E3 | UkrTet8 | 1 | 0.938 | 0.333 | ns |
| E3 | UkrTet9 | 15 | 6.771 | 0.964 | ns |
| E4 | UkrTet1 | 45 | 30.407 | 0.953 | ns |
| E4 | UkrTet3 | 66 | 62.129 | 0.612 | ns |
| E4 | UkrTet4 | 15 | 18.38 | 0.243 | ns |
| E4 | UkrTet5 | 10 | 4.878 | 0.899 | ns |
| E4 | UkrTet6 | 3 | 0.6 | 0.896 | ns |
| E4 | UkrTet7 | 21 | 16.594 | 0.735 | ns |
| E4 | UkrTet8 | 6 | 5.729 | 0.454 | ns |
| E4 | UkrTet9 | 36 | 35.7 | 0.483 | ns |
| E5 | UkrTet1 | 15 | 20 | 0.172 | ns |
| E5 | UkrTet3 | 6 | 2.689 | 0.847 | ns |
| E5 | UkrTet4 | 10 | 2.222 | 0.994 | ns |
| E5 | UkrTet5 | 3 | 2.222 | 0.528 | ns |
| E5 | UkrTet6 | 6 | 0.918 | 0.989 | ns |
| E5 | UkrTet7 | 6 | 2.222 | 0.898 | ns |
| E5 | UkrTet8 | 3 | 2.45 | 0.484 | ns |
| E5 | UkrTet9 | 15 | 8.75 | 0.89 | ns |
| E6 | UkrTet1 | 55 | 51.333 | 0.616 | ns |
| E6 | UkrTet3 | 45 | 33.467 | 0.897 | ns |
| E6 | UkrTet4 | 10 | 8.259 | 0.604 | ns |
| E6 | UkrTet5 | 28 | 33.833 | 0.206 | ns |
| E6 | UkrTet6 | 6 | 5.387 | 0.495 | ns |
| E6 | UkrTet7 | 15 | 15.053 | 0.448 | ns |
| E6 | UkrTet8 | 6 | 2.963 | 0.813 | ns |
| E6 | UkrTet9 | 45 | 46 | 0.431 | ns |
| E7 | UkrTet1 | 45 | 61.401 | 0.052 | ns |
| E7 | UkrTet3 | 55 | 55.391 | 0.46 | ns |
| E7 | UkrTet4 | 21 | 20.829 | 0.469 | ns |
| E7 | UkrTet5 | 28 | 20.211 | 0.857 | ns |
| E7 | UkrTet6 | 3 | 3.921 | 0.27 | ns |
| E7 | UkrTet7 | 15 | 14.02 | 0.524 | ns |
| E7 | UkrTet8 | 6 | 5.6 | 0.469 | ns |
| E7 | UkrTet9 | 28 | 13.62 | 0.99 | ns |
| E8 | UkrTet1 | 3 | 11.479 | 0.009 | ** |
| E8 | UkrTet3 | 1 | 0.098 | 0.754 | ns |
| E8 | UkrTet4 | 1 | 0.311 | 0.577 | ns |
| E8 | UkrTet5 | 3 | 4.014 | 0.26 | ns |
| E8 | UkrTet6 | 1 | 10 | 0.002 | ** |
| E8 | UkrTet7 | 3 | 10 | 0.019 | * |
| E8 | UkrTet8 | 3 | 0.311 | 0.958 | ns |
| E8 | UkrTet9 | 6 | 10 | 0.125 | ns |
| F1 | UkrTet1 | 36 | 40.834 | 0.266 | ns |
| F1 | UkrTet3 | 66 | 54.583 | 0.841 | ns |
| F1 | UkrTet4 | 21 | 21.565 | 0.425 | ns |
| F1 | UkrTet5 | 21 | 24.36 | 0.276 | ns |
| F1 | UkrTet6 | 3 | 0.6 | 0.896 | ns |
| F1 | UkrTet7 | 10 | 6.739 | 0.75 | ns |
| F1 | UkrTet8 | 3 | 1.871 | 0.6 | ns |
| F1 | UkrTet9 | 36 | 22.741 | 0.958 | ns |
| F2 | UkrTet1 | 15 | 11.8 | 0.694 | ns |
| F2 | UkrTet3 | 10 | 8.75 | 0.556 | ns |
| F2 | UkrTet4 | 6 | 6.667 | 0.353 | ns |
| F2 | UkrTet5 | 6 | 5 | 0.544 | ns |
| F2 | UkrTet6 | 3 | 2.361 | 0.501 | ns |
| F2 | UkrTet7 | 3 | 0.918 | 0.821 | ns |
| F2 | UkrTet8 | 6 | 3.75 | 0.71 | ns |
| F2 | UkrTet9 | 21 | 18.333 | 0.628 | ns |
| F3 | UkrTet1 | 91 | 94.167 | 0.389 | ns |
| F3 | UkrTet3 | 91 | 113.25 | 0.057 | ns |
| F3 | UkrTet4 | 21 | 15.763 | 0.783 | ns |
| F3 | UkrTet5 | 15 | 10.269 | 0.803 | ns |
| F3 | UkrTet6 | 1 | 2.269 | 0.132 | ns |
| F3 | UkrTet7 | 10 | 4.934 | 0.896 | ns |
| F3 | UkrTet8 | 10 | 4.32 | 0.932 | ns |
| F3 | UkrTet9 | 78 | 71.55 | 0.684 | ns |
| F4 | UkrTet1 | 36 | 41.583 | 0.241 | ns |
| F4 | UkrTet3 | 55 | 53.775 | 0.522 | ns |
| F4 | UkrTet4 | 15 | 10.232 | 0.805 | ns |
| F4 | UkrTet5 | 21 | 26.592 | 0.185 | ns |
| F4 | UkrTet6 | 6 | 9.055 | 0.171 | ns |
| F4 | UkrTet7 | 15 | 13.8 | 0.541 | ns |
| F4 | UkrTet8 | 6 | 4.477 | 0.612 | ns |
| F4 | UkrTet9 | 36 | 38.438 | 0.36 | ns |
| G1 | UkrTet1 | 45 | 29.375 | 0.965 | ns |
| G1 | UkrTet3 | 28 | 30.555 | 0.337 | ns |
| G1 | UkrTet4 | 21 | 12.788 | 0.916 | ns |
| G1 | UkrTet5 | 6 | 8.038 | 0.235 | ns |
| G1 | UkrTet6 | Monomorphic | |  |  |
| G1 | UkrTet7 | 6 | 7.5 | 0.277 | ns |
| G1 | UkrTet8 | 15 | 10.279 | 0.802 | ns |
| G1 | UkrTet9 | 28 | 45.233 | 0.021 | * |
| G2 | UkrTet1 | 15 | 15.5 | 0.416 | ns |
| G2 | UkrTet3 | 21 | 32.449 | 0.053 | ns |
| G2 | UkrTet4 | 10 | 7.517 | 0.676 | ns |
| G2 | UkrTet5 | 3 | 8.704 | 0.034 | * |
| G2 | UkrTet6 | Monomorphic | |  |  |
| G2 | UkrTet7 | 3 | 19.224 | 0 | *** |
| G2 | UkrTet8 | 6 | 3.022 | 0.806 | ns |
| G2 | UkrTet9 | 15 | 13.35 | 0.575 | ns |
| G3 | UkrTet1 | 66 | 47.6 | 0.957 | ns |
| G3 | UkrTet3 | 66 | 39.252 | 0.996 | ns |
| G3 | UkrTet4 | 36 | 20.079 | 0.985 | ns |
| G3 | UkrTet5 | 15 | 17.274 | 0.303 | ns |
| G3 | UkrTet6 | Monomorphic | |  |  |
| G3 | UkrTet7 | 21 | 14.642 | 0.84 | ns |
| G3 | UkrTet8 | 10 | 4.517 | 0.921 | ns |
| G3 | UkrTet9 | 15 | 27 | 0.029 | * |
| G4 | UkrTet1 | 55 | 54.625 | 0.489 | ns |
| G4 | UkrTet3 | 45 | 34.855 | 0.862 | ns |
| G4 | UkrTet4 | 36 | 46.588 | 0.111 | ns |
| G4 | UkrTet5 | 15 | 13.071 | 0.597 | ns |
| G4 | UkrTet6 | 1 | 0.15 | 0.699 | ns |
| G4 | UkrTet7 | 36 | 40.57 | 0.276 | ns |
| G4 | UkrTet8 | 10 | 5.154 | 0.881 | ns |
| G4 | UkrTet9 | 15 | 7.586 | 0.939 | ns |
| G5 | UkrTet1 | 45 | 61.875 | 0.048 | * |
| G5 | UkrTet3 | 66 | 52.36 | 0.889 | ns |
| G5 | UkrTet4 | 55 | 48.278 | 0.727 | ns |
| G5 | UkrTet5 | 10 | 7.936 | 0.635 | ns |
| G5 | UkrTet6 | 1 | 1.547 | 0.214 | ns |
| G5 | UkrTet7 | 28 | 23.422 | 0.712 | ns |
| G5 | UkrTet8 | 6 | 6.951 | 0.325 | ns |
| G5 | UkrTet9 | 10 | 11.362 | 0.33 | ns |
| H1 | UkrTet1 | 15 | 18.021 | 0.262 | ns |
| H1 | UkrTet3 | 45 | 27.65 | 0.98 | ns |
| H1 | UkrTet4 | 3 | 1.65 | 0.648 | ns |
| H1 | UkrTet5 | 10 | 5.296 | 0.871 | ns |
| H1 | UkrTet6 | Monomorphic | |  |  |
| H1 | UkrTet7 | 6 | 5.612 | 0.468 | ns |
| H1 | UkrTet8 | 1 | 1.519 | 0.218 | ns |
| H1 | UkrTet9 | 3 | 3.046 | 0.385 | ns |
| H2 | UkrTet1 | 21 | 12.153 | 0.935 | ns |
| H2 | UkrTet3 | 21 | 29.388 | 0.105 | ns |
| H2 | UkrTet4 | 10 | 6.883 | 0.736 | ns |
| H2 | UkrTet5 | 6 | 5.833 | 0.442 | ns |
| H2 | UkrTet6 | Monomorphic | |  |  |
| H2 | UkrTet7 | 6 | 1.837 | 0.934 | ns |
| H2 | UkrTet8 | 3 | 0.725 | 0.867 | ns |
| H2 | UkrTet9 | 36 | 37.378 | 0.406 | ns |
|  |  |  |  |  |  |
| Key: ns=not significant, * P<0.05, ** P<0.01, *** P<0.001 | | | | | |
