# Supplementary material for: Population Genetic Patterns of Threatened European Mudminnow (Umbra krameri Walbaum, 1792) in a Fragmented Landscape: Implications for Conservation Management
Source: PLoS One. 2015 Sep 22;10(9):e0138640. doi: 10.1371/journal.pone.0138640 (PMC4578892; doi:10.1371/journal.pone.0138640)
Supplement: S2 Table — (DOCX) [file pone.0138640.s002.docx]

S2 Table

| Microsatellite data of Carpathian Umbra stocks (404individuals, 33populations, 8regions) | | | | | | | | | |
| --- | --- | --- | --- | --- | --- | --- | --- | --- | --- |
| UkrTet1 |  |  |  |  |  |  |  |  |  |
| UkrTet3 |  |  |  |  |  |  |  |  |  |
| UkrTet4 |  |  |  |  |  |  |  |  |  |
| UkrTet5 |  |  |  |  |  |  |  |  |  |
| UkrTet6 |  |  |  |  |  |  |  |  |  |
| UkrTet7 |  |  |  |  |  |  |  |  |  |
| UkrTet8 |  |  |  |  |  |  |  |  |  |
| UkrTet9 |  |  |  |  |  |  |  |  |  |
| Pop |  |  |  |  |  |  |  |  |  |
| A1_U35 | , | 171171 | 159171 | 129133 | 278286 | 229233 | 232252 | 186206 | 253265 |
| A1_U36 | , | 131159 | 159215 | 125141 | 274282 | 229233 | 228252 | 186186 | 249249 |
| A1_U37 | , | 131171 | 171183 | 133145 | 278278 | 229229 | 224228 | 186186 | 253269 |
| A1_U38 | , | 131179 | 159199 | 133189 | 274278 | 229229 | 224236 | 186194 | 253269 |
| A1_U39 | , | 167183 | 179203 | 129137 | 274278 | 233233 | 220248 | 186186 | 269269 |
| A1_U40 | , | 131171 | 187207 | 133133 | 270286 | 229233 | 224232 | 186186 | 249265 |
| A1_U41 | , | 159171 | 199211 | 133197 | 270278 | 229229 | 220220 | 186206 | 257261 |
| A1_U42 | , | 159159 | 163211 | 129145 | 278282 | 229233 | 224252 | 210222 | 249269 |
| A1_U43 | , | 131171 | 159195 | 137189 | 278278 | 229229 | 232252 | 186194 | 249281 |
| A1_U44 | , | 159163 | 171179 | 129141 | 274278 | 229229 | 256256 | 186194 | 249249 |
| A1_U45 | , | 131163 | 183199 | 125193 | 270278 | 229229 | 232256 | 186186 | 249249 |
| A1_U46 | , | 131179 | 159211 | 129185 | 282282 | 229229 | 232236 | 190194 | 265269 |
| A1_U47 | , | 171179 | 159207 | 125141 | 274282 | 229229 | 220252 | 186194 | 265265 |
| A1_U48 | , | 159167 | 151183 | 125129 | 282286 | 229233 | 224252 | 186194 | 249253 |
| Pop |  |  |  |  |  |  |  |  |  |
| A2_U255 | , | 135171 | 151171 | 125137 | 278282 | 229233 | 228252 | 194222 | 249249 |
| A2_U256 | , | 159163 | 199207 | 125133 | 270286 | 229229 | 228228 | 190194 | 253253 |
| A2_U257 | , | 167179 | 171191 | 137145 | 270290 | 229229 | 212212 | 210218 | 265269 |
| A2_U258 | , | 139171 | 187195 | 133145 | 286286 | 229229 | 212252 | 190222 | 261265 |
| A2_U259 | , | 159171 | 163207 | 125145 | 278286 | 229229 | 228232 | 194194 | 265269 |
| A2_U260 | , | 139171 | 175187 | 125133 | 270282 | 229229 | 212228 | 186222 | 253269 |
| A2_U261 | , | 159159 | 151179 | 129145 | 270278 | 229229 | 212228 | 190190 | 249269 |
| A2_U262 | , | 139167 | 151187 | 133133 | 278282 | 229229 | 228252 | 222222 | 249249 |
| A2_U263 | , | 135171 | 159203 | 137145 | 278286 | 229229 | 212228 | 222222 | 265269 |
| A2_U264 | , | 171179 | 171203 | 125145 | 278282 | 229229 | 212212 | 218222 | 249269 |
| A2_U265 | , | 167171 | 159187 | 133145 | 270278 | 229229 | 212248 | 182194 | 253313 |
| A2_U266 | , | 171183 | 163207 | 145145 | 270278 | 229229 | 228228 | 186186 | 265269 |
| Pop |  |  |  |  |  |  |  |  |  |
| A3_P31 | , | 131151 | 171187 | 133141 | 270278 | 229233 | 224228 | 182194 | 257285 |
| A3_P32 | , | 139147 | 171211 | 141141 | 270278 | 229229 | 220228 | 190194 | 245281 |
| A3_P33 | , | 131163 | 179211 | 137137 | 278278 | 229233 | 220228 | 182194 | 265293 |
| A3_P34 | , | 139155 | 171211 | 133137 | 278282 | 229229 | 220220 | 190206 | 253301 |
| A3_P35 | , | 131151 | 171195 | 137141 | 274278 | 229229 | 212224 | 182194 | 253285 |
| Pop |  |  |  |  |  |  |  |  |  |
| A4_P40 | , | 147159 | 171195 | 133133 | 270278 | 229229 | 212224 | 182182 | 285301 |
| A4_P41 | , | 131151 | 195207 | 133137 | 270278 | 229229 | 212216 | 182190 | 245245 |
| A4_P42 | , | 147151 | 171175 | 133137 | 270274 | 229229 | 212216 | 190206 | 245305 |
| A4_P43 | , | 131155 | 195203 | 133137 | 270278 | 229229 | 212212 | 182194 | 253309 |
| A4_P44 | , | 147163 | 171195 | 137137 | 270278 | 229229 | 212220 | 198198 | 249297 |
| A4_P45 | , | 151155 | 195211 | 133137 | 270270 | 229229 | 216220 | 182182 | 245273 |
| A4_P46 | , | 135155 | 171195 | 133141 | 270282 | 229233 | 212216 | 182190 | 245285 |
| A4_P47 | , | 159171 | 195199 | 137141 | 278294 | 229229 | 224224 | 182182 | 281305 |
| A4_P48 | , | 123131 | 151175 | 149149 | 270282 | 229233 | 220224 | 202206 | 261269 |
| A4_P49 | , | 147155 | 175199 | 133133 | 270278 | 229233 | 220224 | 182202 | 245301 |
| A4_P50 | , | 131131 | 191211 | 137137 | 270278 | 229229 | 220224 | 182198 | 245285 |
| A4_P51 | , | 159175 | 171207 | 145145 | 278278 | 229229 | 224224 | 182198 | 245249 |
| A4_P52 | , | 151155 | 195199 | 133133 | 274294 | 229233 | 212212 | 202202 | 245301 |
| A4_P53 | , | 131131 | 151171 | 133141 | 278278 | 229229 | 216228 | 186202 | 265305 |
| A4_P54 | , | 147151 | 171195 | 133137 | 278278 | 229229 | 212212 | 182190 | 249253 |
| Pop |  |  |  |  |  |  |  |  |  |
| B1_U141 | , | 135183 | 171179 | 141145 | 274282 | 229229 | 240240 | 206218 | 249269 |
| B1_U142 | , | 135171 | 183187 | 137137 | 270282 | 229229 | 236240 | 186214 | 253261 |
| B1_U143 | , | 135151 | 175191 | 137173 | 286298 | 229229 | 220228 | 190202 | 261269 |
| B1_U144 | , | 135159 | 183187 | 121133 | 270278 | 229229 | 224236 | 194194 | 241257 |
| B1_U145 | , | 151159 | 191207 | 141149 | 278298 | 229229 | 228232 | 182206 | 253293 |
| B1_U146 | , | 139179 | 183187 | 129137 | 266282 | 229229 | 232232 | 186218 | 269281 |
| B1_U147 | , | 135163 | 167187 | 129137 | 282282 | 229229 | 212232 | 202210 | 241249 |
| B1_U148 | , | 135139 | 187191 | 145145 | 278286 | 229229 | 216224 | 186206 | 249261 |
| B1_U149 | , | 135139 | 187203 | 137137 | 270278 | 229229 | 236244 | 210218 | 249277 |
| B1_U150 | , | 143163 | 191195 | 133141 | 270294 | 229229 | 232236 | 186190 | 249253 |
| B1_U151 | , | 139163 | 147183 | 137169 | 278282 | 229229 | 212220 | 182202 | 249269 |
| B1_U152 | , | 139139 | 147191 | 133137 | 282298 | 229229 | 224244 | 182194 | 269281 |
| B1_U153 | , | 127131 | 183187 | 129149 | 278286 | 229229 | 224228 | 186190 | 249297 |
| B1_U154 | , | 139151 | 187191 | 125141 | 278290 | 229233 | 216244 | 198218 | 249253 |
| B1_U155 | , | 139163 | 187191 | 129129 | 278290 | 229233 | 216244 | 182182 | 253253 |
| Pop |  |  |  |  |  |  |  |  |  |
| B2_P30 | , | 135159 | 183183 | 129137 | 270298 | 229229 | 236244 | 182194 | 269273 |
| B2_U157 | , | 163175 | 159175 | 133145 | 278294 | 229229 | 224232 | 194214 | 253261 |
| B2_U158 | , | 123131 | 159203 | 129149 | 278290 | 229229 | 220224 | 182226 | 253253 |
| B2_U159 | , | 123171 | 175183 | 145145 | 274286 | 229229 | 220240 | 178206 | 249281 |
| B2_U160 | , | 131143 | 155171 | 125149 | 266274 | 229229 | 224240 | 190194 | 253289 |
| B2_U161 | , | 151159 | 167171 | 121149 | 294294 | 229229 | 240240 | 190214 | 253285 |
| B2_U162 | , | 135171 | 191207 | 137145 | 270274 | 229229 | 220232 | 198214 | 249257 |
| B2_U163 | , | 139151 | 179187 | 133137 | 274278 | 229229 | 220232 | 182186 | 249249 |
| B2_U164 | , | 143147 | 191207 | 137137 | 274290 | 229229 | 232248 | 194198 | 273285 |
| B2_U165 | , | 143179 | 183199 | 145161 | 270270 | 229229 | 224236 | 194222 | 257269 |
| B2_U166 | , | 163179 | 147175 | 129157 | 286294 | 233233 | 220220 | 186222 | 241261 |
| B2_U167 | , | 127163 | 191199 | 141141 | 282298 | 229233 | 224228 | 186190 | 249305 |
| B2_U168 | , | 123171 | 191195 | 133133 | 274278 | 229229 | 216220 | 194194 | 265269 |
| B2_U169 | , | 151175 | 195195 | 137137 | 274278 | 229229 | 220236 | 186194 | 253257 |
| B2_U170 | , | 147151 | 147163 | 137137 | 270274 | 229229 | 216244 | 186190 | 253285 |
| Pop |  |  |  |  |  |  |  |  |  |
| B3_PP1 | , | 147163 | 163167 | 137149 | 274282 | 229229 | 220224 | 190190 | 241265 |
| B3_PP2 | , | 139143 | 147167 | 125133 | 274278 | 229233 | 216220 | 214218 | 241253 |
| B3_PP3 | , | 131131 | 187187 | 125141 | 278282 | 229233 | 236244 | 190190 | 253253 |
| B3_PP4 | , | 155163 | 195199 | 133141 | 278282 | 229229 | 220224 | 186186 | 253301 |
| B3_PP5 | , | 127163 | 179199 | 133145 | 282282 | 229229 | 224244 | 186214 | 241301 |
| B3_PP6 | , | 143143 | 147175 | 137141 | 278278 | 229229 | 220244 | 190190 | 245301 |
| B3_PP7 | , | 167167 | 179199 | 133145 | 274278 | 229229 | 220236 | 174214 | 253301 |
| B3_PP8 | , | 127127 | 151151 | 133149 | 278282 | 229229 | 216252 | 186206 | 253301 |
| B3_PP9 | , | 143159 | 147195 | 133137 | 278282 | 229229 | 220232 | 186206 | 253253 |
| B3_PP10 | , | 131143 | 163199 | 133137 | 274278 | 229229 | 216220 | 190190 | 253265 |
| Pop |  |  |  |  |  |  |  |  |  |
| C1_T01 | , | 143143 | 207227 | 121145 | 266266 | 229229 | 236236 | 178182 | 277281 |
| C1_T02 | , | 143151 | 207215 | 121145 | 266270 | 229229 | 228240 | 182186 | 289289 |
| C1_T03 | , | 143143 | 211223 | 141141 | 266270 | 229229 | 240240 | 182182 | 277289 |
| C1_T04 | , | 143143 | 207207 | 141145 | 270286 | 229229 | 228240 | 178182 | 277289 |
| C1_T05 | , | 143143 | 219223 | 141145 | 270286 | 229229 | 236240 | 182182 | 281289 |
| Pop |  |  |  |  |  |  |  |  |  |
| C2_M01 | , | 143151 | 211211 | 121121 | 266266 | 229229 | 228240 | 182182 | 289289 |
| C2_M02 | , | 143143 | 207215 | 141145 | 270286 | 229229 | 240240 | 182182 | 277277 |
| C2_M03 | , | 143159 | 207215 | 141141 | 266286 | 229229 | 236236 | 182182 | 277281 |
| C2_M04 | , | 143151 | 207211 | 141145 | 266286 | 229229 | 240240 | 182182 | 289289 |
| C2_M05 | , | 143143 | 207219 | 141141 | 266266 | 229229 | 240240 | 182182 | 281289 |
| C2_M06 | , | 139159 | 207223 | 141145 | 266266 | 229229 | 236240 | 178182 | 277289 |
| C2_M07 | , | 143151 | 215223 | 145145 | 286286 | 229229 | 228236 | 178182 | 277289 |
| C2_M08 | , | 143143 | 215231 | 121141 | 266286 | 229229 | 232236 | 182182 | 289289 |
| C2_M09 | , | 143143 | 211215 | 141141 | 266286 | 229229 | 236240 | 182182 | 281289 |
| C2_M10 | , | 143143 | 211215 | 141141 | 266286 | 229229 | 236240 | 182182 | 277281 |
| C2_M11 | , | 143159 | 211223 | 121141 | 266286 | 229229 | 240240 | 178182 | 277289 |
| C2_M12 | , | 143143 | 211211 | 141145 | 266266 | 229229 | 236240 | 182182 | 277277 |
| C2_M13 | , | 143143 | 211215 | 141141 | 266270 | 229229 | 236240 | 182182 | 277289 |
| C2_M14 | , | 143143 | 203215 | 141141 | 286286 | 229229 | 240240 | 182182 | 277281 |
| C2_M15 | , | 143151 | 207211 | 121141 | 266286 | 229229 | 228240 | 182182 | 277281 |
| Pop |  |  |  |  |  |  |  |  |  |
| D1_U1 | , | 167167 | 167171 | 137137 | 294298 | 229229 | 220220 | 194206 | 245245 |
| D1_U2 | , | 143167 | 167183 | 137137 | 298298 | 229229 | 220220 | 194214 | 289289 |
| D1_U3 | , | 143167 | 171183 | 133133 | 278302 | 229229 | 224224 | 194206 | 245289 |
| D1_U4 | , | 143167 | 167183 | 133137 | 294298 | 229229 | 220224 | 194194 | 245289 |
| D1_U5 | , | 143143 | 167183 | 133133 | 298298 | 229229 | 224224 | 194194 | 245289 |
| D1_U6 | , | 143143 | 167171 | 133133 | 278298 | 229229 | 216216 | 190194 | 281281 |
| D1_U7 | , | 143167 | 171171 | 137137 | 278294 | 229229 | 220224 | 206214 | 245289 |
| D1_U8 | , | 143167 | 183183 | 133137 | 294298 | 229229 | 220224 | 198214 | 245245 |
| D1_U9 | , | 143167 | 167171 | 133137 | 294318 | 229229 | 220220 | 194194 | 245289 |
| D1_U10 | , | 167167 | 167167 | 133133 | 278298 | 229229 | 220220 | 194206 | 245289 |
| D1_U11 | , | 143167 | 167167 | 133133 | 298298 | 229229 | 220220 | 194214 | 245289 |
| D1_U12 | , | 167167 | 167171 | 133137 | 294294 | 229229 | 220224 | 190206 | 281281 |
| D1_U13 | , | 143167 | 167167 | 133137 | 278294 | 229229 | 216220 | 190194 | 245289 |
| D1_U14 | , | 167167 | 183183 | 133137 | 278294 | 229229 | 216220 | 194194 | 245289 |
| D1_U15 | , | 167167 | 167171 | 137137 | 278294 | 229229 | 220220 | 190194 | 245245 |
| Pop |  |  |  |  |  |  |  |  |  |
| D2_A1 | , | 179179 | 175175 | 137141 | 278278 | 229229 | 220244 | 186214 | 285285 |
| D2_A2 | , | 179179 | 175175 | 133137 | 278278 | 229233 | 220224 | 214214 | 285289 |
| D2_A3 | , | 147147 | 175195 | 137137 | 278282 | 229229 | 220220 | 186214 | 297297 |
| D2_A4 | , | 155179 | 167175 | 137137 | 278278 | 229229 | 220220 | 210214 | 285289 |
| D2_A5 | , | 151151 | 175175 | 137141 | 278278 | 229229 | 220220 | 210214 | 293297 |
| D2_A6 | , | 151151 | 175175 | 137137 | 278278 | 229229 | 220220 | 186214 | 289297 |
| D2_A7 | , | 151179 | 175175 | 137141 | 278278 | 229229 | 220244 | 210214 | 285285 |
| D2_A8 | , | 151151 | 167175 | 141145 | 274298 | 229229 | 220220 | 210214 | 285285 |
| D2_A9 | , | 151167 | 179195 | 137141 | 278278 | 229229 | 220220 | 186210 | 285297 |
| D2_A10 | , | 151151 | 175175 | 137137 | 278298 | 229229 | 220220 | 210214 | 285293 |
| Pop |  |  |  |  |  |  |  |  |  |
| D3_K334 | , | 147167 | 167175 | 137137 | 278282 | 229229 | 220224 | 206214 | 285285 |
| D3_K335 | , | 147175 | 167195 | 137137 | 278278 | 229229 | 220220 | 214214 | 285285 |
| D3_K336 | , | 171179 | 175175 | 145169 | 278278 | 229229 | 216220 | 186214 | 281285 |
| D3_K337 | , | 147147 | 175175 | 137137 | 278278 | 229229 | 220220 | 186210 | 285305 |
| D3_K338 | , | 147179 | 167171 | 141141 | 278278 | 229229 | 220244 | 210214 | 245289 |
| D3_K339 | , | 147147 | 171171 | 137169 | 278278 | 229229 | 220220 | 186206 | 285305 |
| D3_K340 | , | 147179 | 171175 | 133145 | 278278 | 229229 | 220220 | 210214 | 285289 |
| D3_K341 | , | 147151 | 167175 | 137137 | 266274 | 229229 | 220244 | 186206 | 285285 |
| D3_K342 | , | 147167 | 175175 | 137141 | 278282 | 229229 | 220220 | 214214 | 289293 |
| D3_K343 | , | 147179 | 171175 | 133137 | 278278 | 229229 | 220220 | 186186 | 293293 |
| Pop |  |  |  |  |  |  |  |  |  |
| D4_K344 | , | 187207 | 179183 | 133141 | 266282 | 229229 | 248256 | 186186 | 257257 |
| D4_K345 | , | 187207 | 159175 | 133165 | 270290 | 229229 | 216228 | 190202 | 245297 |
| D4_K346 | , | 139171 | 171211 | 137149 | 274286 | 229229 | 220224 | 202206 | 269305 |
| D4_K347 | , | 171187 | 199211 | 161165 | 266278 | 229229 | 248252 | 194202 | 245269 |
| D4_K348 | , | 135151 | 175199 | 149153 | 266274 | 229229 | 216236 | 186194 | 241253 |
| D4_K349 | , | 139207 | 179183 | 133141 | 266278 | 225229 | 232248 | 186194 | 245269 |
| D4_K350 | , | 187207 | 175183 | 141149 | 274290 | 229229 | 216236 | 182194 | 245273 |
| D4_K351 | , | 159175 | 175175 | 133149 | 274290 | 229229 | 216220 | 186186 | 273273 |
| D4_K352 | , | 187207 | 171175 | 133145 | 290274 | 229229 | 216216 | 186222 | 273305 |
| D4_K353 | , | 195195 | 191199 | 137165 | 274274 | 229229 | 220228 | 182186 | 257273 |
| Pop |  |  |  |  |  |  |  |  |  |
| D5_K354 | , | 187207 | 175187 | 149161 | 274274 | 229229 | 224228 | 186190 | 269301 |
| D5_K355 | , | 155155 | 171183 | 137149 | 278290 | 229229 | 248248 | 186190 | 241281 |
| D5_K356 | , | 179183 | 183207 | 137157 | 266274 | 229229 | 220248 | 186218 | 269273 |
| D5_K357 | , | 175195 | 175183 | 161161 | 278278 | 229229 | 248248 | 198198 | 241273 |
| D5_K358 | , | 171203 | 171183 | 149161 | 274274 | 229229 | 224228 | 186198 | 273273 |
| Pop |  |  |  |  |  |  |  |  |  |
| E1_U267 | , | 195195 | 191207 | 133133 | 278298 | 229229 | 208212 | 190198 | 269285 |
| E1_U268 | , | 195207 | 191203 | 133137 | 298298 | 229229 | 212216 | 186190 | 269269 |
| E1_U408 | , | 195199 | 163203 | 133145 | 278298 | 229229 | 212216 | 190190 | 281285 |
| E1_U412 | , | 195195 | 199203 | 137145 | 298298 | 229229 | 216216 | 190190 | 269269 |
| E1_U413 | , | 199203 | 191203 | 133145 | 298298 | 229229 | 208216 | 190190 | 269269 |
| E1_U415 | , | 195195 | 191191 | 137137 | 298298 | 229229 | 212224 | 190190 | 269285 |
| E1_U417 | , | 195195 | 199203 | 133137 | 298298 | 229229 | 212224 | 190190 | 269269 |
| E1_U419 | , | 203211 | 191195 | 133137 | 298298 | 229229 | 212216 | 190190 | 269285 |
| E1_U422 | , | 195195 | 203203 | 137145 | 278298 | 229229 | 212212 | 174190 | 269269 |
| E1_U424 | , | 195195 | 163199 | 133133 | 298298 | 229237 | 216216 | 174190 | 281281 |
| E1_S01 | , | 199207 | 187223 | 133133 | 298298 | 229229 | 216216 | 190190 | 273281 |
| E1_S02 | , | 191207 | 215227 | 133145 | 298298 | 229229 | 212212 | 190190 | 269285 |
| E1_S03 | , | 191191 | 187223 | 133133 | 298298 | 229229 | 216224 | 190190 | 269285 |
| E1_S04 | , | 191199 | 223223 | 133145 | 278298 | 229229 | 216216 | 186190 | 269281 |
| E1_S05 | , | 191199 | 191215 | 133133 | 298298 | 229229 | 208224 | 174174 | 269269 |
| E1_S06 | , | 191199 | 215223 | 137145 | 278298 | 229229 | 208212 | 190198 | 281289 |
| E1_S07 | , | 191207 | 219227 | 133137 | 298298 | 229229 | 212216 | 190190 | 269285 |
| E1_S08 | , | 199203 | 187215 | 133137 | 278278 | 229229 | 212216 | 190190 | 269285 |
| E1_S09 | , | 191199 | 215223 | 133141 | 278298 | 229229 | 216224 | 190190 | 269277 |
| E1_S10 | , | 203203 | 215223 | 133133 | 298298 | 229229 | 212212 | 174174 | 269285 |
| Pop |  |  |  |  |  |  |  |  |  |
| E2_P01 | , | 183191 | 167171 | 153153 | 286310 | 237241 | 220240 | 186186 | 273285 |
| E2_P02 | , | 187187 | 159167 | 137145 | 286286 | 237241 | 224224 | 178186 | 277281 |
| E2_P03 | , | 179183 | 155163 | 145149 | 286310 | 229233 | 236236 | 174186 | 273277 |
| E2_P04 | , | 183195 | 183183 | 149149 | 286286 | 233241 | 220224 | 186186 | 285285 |
| E2_P05 | , | 179191 | 171203 | 149149 | 286286 | 237237 | 220236 | 178186 | 285285 |
| E2_P06 | , | 191199 | 155199 | 149149 | 286286 | 237241 | 232236 | 174186 | 281285 |
| E2_P07 | , | 187187 | 179183 | 137149 | 286286 | 237241 | 220236 | 178186 | 273277 |
| E2_P08 | , | 187191 | 187187 | 149149 | 286310 | 233241 | 220240 | 186186 | 277285 |
| E2_P09 | , | 187191 | 179179 | 133153 | 286310 | 237237 | 224236 | 174186 | 285285 |
| E2_P10 | , | 183191 | 175179 | 133145 | 310310 | 241241 | 220220 | 186186 | 273285 |
| Pop |  |  |  |  |  |  |  |  |  |
| E3_P222 | , | 159183 | 147175 | 145145 | 278278 | 237237 | 220220 | 174178 | 281297 |
| E3_P223 | , | 159183 | 147207 | 145145 | 286286 | 233237 | 216236 | 174178 | 281281 |
| E3_P224 | , | 183183 | 175175 | 141149 | 286290 | 229237 | 216232 | 174174 | 285289 |
| E3_P225 | , | 159171 | 179203 | 141145 | 278278 | 237237 | 216236 | 174174 | 281285 |
| E3_P226 | , | 159171 | 203207 | 141145 | 286290 | 237237 | 212220 | 174174 | 281297 |
| E3_P227 | , | 163163 | 183203 | 141145 | 282286 | 237237 | 216220 | 174174 | 277285 |
| E3_P228 | , | 167167 | 183199 | 145145 | 286290 | 237237 | 232236 | 174174 | 289297 |
| E3_P229 | , | 171183 | 179187 | 145153 | 286290 | 229237 | 216236 | 174178 | 281293 |
| E3_P230 | , | 183183 | 151207 | 129141 | 278278 | 237237 | 212236 | 174178 | 281281 |
| E3_P231 | , | 159159 | 199207 | 145157 | 286290 | 237237 | 212220 | 174174 | 277281 |
| E3_P232 | , | 179191 | 147211 | 145145 | 278286 | 237237 | 216220 | 174178 | 277277 |
| E3_P233 | , | 159159 | 207207 | 141141 | 282290 | 233237 | 212220 | 174174 | 285297 |
| E3_P234 | , | 159159 | 183203 | 141149 | 278290 | 229237 | 236236 | 174178 | 277281 |
| E3_P235 | , | 171187 | 179187 | 137145 | 278278 | 237237 | 220220 | 174174 | 281285 |
| E3_P236 | , | 151167 | 175183 | 145145 | 278278 | 229237 | 220232 | 174174 | 277289 |
| Pop |  |  |  |  |  |  |  |  |  |
| E4_U171 | , | 171175 | 179191 | 141153 | 282290 | 237237 | 220220 | 174178 | 297301 |
| E4_U172 | , | 175191 | 179179 | 141149 | 282286 | 229237 | 236240 | 174178 | 277293 |
| E4_U173 | , | 167171 | 175207 | 141145 | 282290 | 237237 | 216220 | 174178 | 281305 |
| E4_U174 | , | 163171 | 179207 | 141161 | 278286 | 237237 | 220220 | 178178 | 277281 |
| E4_U175 | , | 171179 | 175179 | 145145 | 290290 | 233237 | 212236 | 174178 | 277281 |
| E4_U176 | , | 187199 | 167195 | 145145 | 278290 | 229237 | 212232 | 178178 | 285289 |
| E4_U177 | , | 159163 | 155199 | 141145 | 278282 | 233237 | 212220 | 174178 | 277277 |
| E4_U178 | , | 159171 | 171203 | 149161 | 278290 | 237237 | 220224 | 174178 | 281297 |
| E4_U179 | , | 171187 | 179203 | 141141 | 278286 | 237237 | 212236 | 178182 | 277277 |
| E4_U180 | , | 159187 | 179203 | 141157 | 286290 | 237237 | 216220 | 174174 | 273285 |
| E4_U181 | , | 171171 | 199211 | 145145 | 286290 | 237237 | 212232 | 174178 | 285285 |
| E4_U182 | , | 187199 | 175203 | 145145 | 290290 | 229237 | 220232 | 178186 | 277289 |
| E4_U183 | , | 167187 | 203207 | 141149 | 286290 | 237237 | 212224 | 174178 | 277281 |
| E4_U184 | , | 171183 | 171195 | 145145 | 286310 | 237237 | 212220 | 174178 | 285293 |
| E4_U185 | , | 159191 | 183195 | 149161 | 278278 | 237237 | 220236 | 174178 | 277289 |
| Pop |  |  |  |  |  |  |  |  |  |
| E5_P242 | , | 199199 | 179199 | 145149 | 286290 | 233237 | 212228 | 178186 | 273281 |
| E5_P243 | , | 171191 | 179195 | 145145 | 286286 | 237237 | 212228 | 174174 | 289301 |
| E5_P244 | , | 195203 | 171179 | 129145 | 286290 | 237241 | 212220 | 174174 | 281285 |
| E5_P245 | , | 167171 | 199199 | 141145 | 286310 | 237237 | 212224 | 178178 | 281289 |
| E5_P246 | , | 195203 | 179179 | 145153 | 286290 | 229237 | 212212 | 174178 | 277281 |
| Pop |  |  |  |  |  |  |  |  |  |
| E6_P192 | , | 175199 | 187199 | 133137 | 282290 | 229237 | 212228 | 186186 | 277281 |
| E6_P193 | , | 151159 | 183191 | 129133 | 282290 | 229241 | 212224 | 182186 | 277305 |
| E6_P194 | , | 163163 | 179203 | 129133 | 286290 | 237237 | 212220 | 182186 | 285309 |
| E6_P195 | , | 171179 | 171191 | 129137 | 290290 | 237237 | 224224 | 174186 | 289293 |
| E6_P196 | , | 147159 | 167207 | 141149 | 278282 | 237237 | 228240 | 174174 | 265281 |
| E6_P197 | , | 155163 | 187203 | 133133 | 290314 | 241241 | 236236 | 178186 | 277305 |
| E6_P198 | , | 163179 | 175187 | 133141 | 282290 | 233237 | 220236 | 174182 | 273305 |
| E6_P199 | , | 171199 | 191203 | 129137 | 286290 | 237241 | 228236 | 174186 | 277321 |
| E6_P200 | , | 151179 | 167191 | 133133 | 290318 | 229229 | 212224 | 182186 | 289305 |
| E6_P201 | , | 171187 | 179199 | 129141 | 286290 | 241241 | 220236 | 174174 | 277277 |
| E6_P202 | , | 163163 | 179191 | 137149 | 294306 | 241241 | 228228 | 174186 | 273305 |
| E6_P203 | , | 179191 | 175179 | 129133 | 290290 | 237241 | 212228 | 174178 | 305309 |
| Pop |  |  |  |  |  |  |  |  |  |
| E7_P176 | , | 175179 | 191207 | 129133 | 278286 | 237237 | 228228 | 178186 | 281305 |
| E7_P177 | , | 163163 | 199199 | 129137 | 286286 | 229237 | 228232 | 182186 | 305313 |
| E7_P178 | , | 163175 | 175195 | 129133 | 282286 | 237237 | 224228 | 174186 | 277305 |
| E7_P179 | , | 175175 | 191199 | 133145 | 286286 | 237241 | 228232 | 174182 | 281293 |
| E7_P180 | , | 163183 | 183199 | 137157 | 278282 | 229241 | 212232 | 178182 | 277305 |
| E7_P181 | , | 151155 | 163179 | 133141 | 286294 | 237237 | 224232 | 174182 | 281305 |
| E7_P182 | , | 171199 | 195199 | 133153 | 290306 | 237241 | 212240 | 178182 | 305305 |
| E7_P183 | , | 167207 | 203203 | 133157 | 286310 | 237241 | 228236 | 174178 | 265281 |
| E7_P184 | , | 163171 | 183207 | 129133 | 278278 | 237241 | 228236 | 174174 | 277281 |
| E7_P185 | , | 175179 | 167199 | 133157 | 286286 | 237237 | 228240 | 174174 | 281285 |
| E7_P186 | , | 163175 | 183191 | 129137 | 282290 | 237237 | 228236 | 174174 | 265281 |
| E7_P187 | , | 175199 | 191195 | 129133 | 286310 | 229241 | 236236 | 174174 | 281305 |
| E7_P188 | , | 179179 | 191199 | 129133 | 278286 | 237237 | 228228 | 174174 | 281305 |
| E7_P189 | , | 163167 | 191203 | 141157 | 290298 | 237237 | 224232 | 174178 | 277281 |
| E7_P190 | , | 175179 | 179187 | 141157 | 286290 | 237241 | 212224 | 178186 | 281317 |
| Pop |  |  |  |  |  |  |  |  |  |
| E8_P248 | , | 187187 | 171171 | 145149 | 286286 | 237241 | 220224 | 186186 | 281285 |
| E8_P249 | , | 187187 | 171175 | 145145 | 282310 | 237241 | 220224 | 186186 | 281285 |
| E8_P250 | , | 175187 | 171171 | 145149 | 282286 | 237241 | 220224 | 186186 | 273281 |
| E8_P251 | , | 187187 | 171175 | 145145 | 282310 | 237241 | 220224 | 186186 | 281285 |
| E8_P252 | , | 187187 | 171175 | 145145 | 282286 | 237241 | 220236 | 178186 | 273281 |
| E8_P253 | , | 175187 | 171175 | 145145 | 286310 | 237241 | 220236 | 178186 | 281285 |
| E8_P254 | , | 175187 | 171171 | 145149 | 282310 | 237241 | 220224 | 186186 | 273281 |
| E8_P255 | , | 175187 | 171171 | 145145 | 282310 | 237241 | 220224 | 186186 | 273281 |
| E8_P256 | , | 175187 | 171175 | 145145 | 286310 | 237241 | 220236 | 186186 | 281285 |
| E8_P257 | , | 183183 | 175175 | 145145 | 282286 | 237241 | 220236 | 174186 | 277281 |
| Pop |  |  |  |  |  |  |  |  |  |
| F1_P101 | , | 175179 | 179187 | 137141 | 282286 | 229237 | 220220 | 174174 | 281289 |
| F1_P102 | , | 187187 | 163179 | 133145 | 282282 | 229237 | 220220 | 166174 | 265289 |
| F1_P103 | , | 179195 | 167199 | 133133 | 282294 | 229229 | 228236 | 174174 | 277285 |
| F1_P104 | , | 159187 | 167183 | 133133 | 298302 | 229237 | 220228 | 166178 | 289305 |
| F1_P105 | , | 167167 | 175195 | 133133 | 278286 | 237237 | 220232 | 166174 | 289293 |
| F1_P106 | , | 167171 | 167179 | 129141 | 278278 | 237237 | 220232 | 174174 | 273289 |
| F1_P107 | , | 171171 | 179195 | 125141 | 282286 | 229241 | 220228 | 174174 | 265273 |
| F1_P108 | , | 171175 | 163183 | 133141 | 282302 | 237237 | 212228 | 174178 | 277305 |
| F1_P109 | , | 187195 | 159191 | 133141 | 278286 | 237237 | 220220 | 174174 | 285289 |
| F1_P110 | , | 195195 | 159163 | 125133 | 286286 | 237237 | 220228 | 166174 | 289289 |
| F1_P111 | , | 175175 | 167191 | 133145 | 278278 | 237237 | 212220 | 166174 | 269289 |
| F1_P112 | , | 191195 | 151207 | 133137 | 282290 | 237241 | 228228 | 166174 | 285305 |
| F1_P113 | , | 195195 | 179199 | 129141 | 282282 | 229237 | 212220 | 174174 | 273281 |
| F1_P114 | , | 167187 | 179191 | 125149 | 282286 | 237237 | 212220 | 166174 | 289289 |
| F1_P115 | , | 183183 | 175207 | 133133 | 282286 | 229237 | 212220 | 174174 | 265293 |
| Pop |  |  |  |  |  |  |  |  |  |
| F2_P131 | , | 159187 | 187191 | 137141 | 282302 | 237241 | 220232 | 178186 | 285289 |
| F2_P132 | , | 183191 | 163167 | 133137 | 282302 | 237237 | 220228 | 166174 | 281289 |
| F2_P133 | , | 175187 | 163191 | 133137 | 278282 | 229237 | 220232 | 174178 | 265285 |
| F2_P134 | , | 167187 | 187191 | 133145 | 282294 | 229229 | 220220 | 174178 | 277293 |
| F2_P135 | , | 187187 | 191195 | 133145 | 278282 | 241241 | 220220 | 166178 | 269289 |
| Pop |  |  |  |  |  |  |  |  |  |
| F3_P141 | , | 183183 | 187191 | 133137 | 282286 | 229237 | 236236 | 174186 | 265293 |
| F3_P142 | , | 151203 | 191211 | 133153 | 278282 | 229229 | 212228 | 178178 | 269273 |
| F3_P143 | , | 183183 | 175207 | 129137 | 278298 | 229237 | 216228 | 174174 | 281321 |
| F3_P144 | , | 179203 | 195211 | 133133 | 282282 | 229229 | 220228 | 182186 | 261285 |
| F3_P145 | , | 163195 | 187187 | 133145 | 282286 | 229237 | 212212 | 174178 | 289297 |
| F3_P146 | , | 183195 | 167171 | 137145 | 278286 | 229237 | 212236 | 174190 | 277293 |
| F3_P147 | , | 179223 | 167207 | 125137 | 282282 | 229237 | 212228 | 174178 | 261293 |
| F3_P148 | , | 167207 | 167171 | 129133 | 278278 | 237237 | 212216 | 174182 | 257273 |
| F3_P149 | , | 175187 | 187191 | 129153 | 274278 | 229237 | 212216 | 174182 | 277281 |
| F3_P150 | , | 199199 | 167203 | 125133 | 278286 | 229229 | 212220 | 174182 | 265289 |
| F3_P151 | , | 191199 | 167171 | 129137 | 282282 | 229237 | 212216 | 174174 | 265293 |
| F3_P152 | , | 183207 | 159175 | 121153 | 278286 | 229229 | 216216 | 174178 | 273277 |
| F3_P153 | , | 191207 | 183191 | 129133 | 282282 | 229237 | 212212 | 174182 | 285293 |
| F3_P154 | , | 175191 | 179199 | 133145 | 282290 | 229237 | 216236 | 174186 | 265277 |
| F3_P155 | , | 171179 | 147183 | 125153 | 278290 | 229237 | 212236 | 178182 | 265313 |
| Pop |  |  |  |  |  |  |  |  |  |
| F4_P161 | , | 187199 | 147179 | 133137 | 278282 | 229237 | 216232 | 174178 | 285293 |
| F4_P162 | , | 179191 | 147187 | 129133 | 278282 | 229233 | 212232 | 174182 | 265281 |
| F4_P163 | , | 167179 | 187191 | 133137 | 282282 | 233237 | 224232 | 174174 | 269281 |
| F4_P164 | , | 175199 | 163171 | 133161 | 278278 | 229229 | 212220 | 174186 | 289289 |
| F4_P165 | , | 167179 | 171187 | 133137 | 278278 | 233237 | 212216 | 174182 | 281297 |
| F4_P166 | , | 175199 | 163171 | 129133 | 282290 | 229237 | 216232 | 174178 | 281297 |
| F4_P167 | , | 179195 | 159171 | 129129 | 282282 | 233237 | 212236 | 178182 | 261285 |
| F4_P168 | , | 167187 | 171191 | 133145 | 278286 | 229229 | 212220 | 174182 | 273281 |
| F4_P169 | , | 191203 | 167175 | 129145 | 278278 | 233237 | 216220 | 174182 | 265269 |
| F4_P170 | , | 167179 | 175183 | 133145 | 282306 | 237245 | 212216 | 182182 | 273289 |
| F4_P171 | , | 195203 | 175207 | 137137 | 278294 | 237237 | 212232 | 174174 | 269285 |
| F4_P172 | , | 191191 | 171171 | 125137 | 278294 | 229229 | 212216 | 182182 | 281285 |
| F4_P173 | , | 167199 | 163171 | 145145 | 278278 | 229229 | 212220 | 174178 | 269281 |
| F4_P174 | , | 167179 | 171171 | 129137 | 282306 | 237245 | 212216 | 182182 | 273289 |
| F4_P175 | , | 171199 | 163187 | 133145 | 298298 | 237237 | 216220 | 174178 | 281297 |
| Pop |  |  |  |  |  |  |  |  |  |
| G1_U238 | , | 155171 | 179195 | 129129 | 282282 | 229229 | 208228 | 182182 | 277285 |
| G1_U239 | , | 187191 | 187195 | 129169 | 282290 | 229229 | 224228 | 182186 | 273277 |
| G1_U240 | , | 163175 | 167187 | 133161 | 290290 | 229229 | 224228 | 182186 | 277277 |
| G1_U241 | , | 175175 | 179199 | 129169 | 282282 | 229229 | 220228 | 182186 | 281301 |
| G1_U242 | , | 167191 | 167179 | 161161 | 282302 | 229229 | 220224 | 178186 | 273277 |
| G1_U243 | , | 167223 | 187195 | 129129 | 282290 | 229229 | 220224 | 182198 | 277285 |
| G1_U244 | , | 155175 | 187187 | 129149 | 286286 | 229229 | 224228 | 182182 | 277285 |
| G1_U245 | , | 223223 | 175183 | 149161 | 282290 | 229229 | 224228 | 190198 | 285297 |
| G1_U246 | , | 191191 | 179199 | 129129 | 282302 | 229229 | 224228 | 182186 | 273285 |
| G1_U247 | , | 171175 | 179195 | 129157 | 282282 | 229229 | 224228 | 186186 | 289293 |
| G1_U248 | , | 155191 | 179195 | 129145 | 290290 | 229229 | 228228 | 182186 | 277297 |
| G1_U249 | , | 151167 | 183187 | 129149 | 282302 | 229229 | 224228 | 186190 | 285289 |
| G1_U250 | , | 175191 | 179187 | 129149 | 282286 | 229229 | 224228 | 182186 | 289297 |
| G1_U251 | , | 175223 | 191195 | 133149 | 290302 | 229229 | 208224 | 186194 | 297297 |
| G1_U252 | , | 175183 | 187195 | 149161 | 282282 | 229229 | 220224 | 186190 | 277277 |
| Pop |  |  |  |  |  |  |  |  |  |
| G2_U121 | , | 187195 | 151199 | 149149 | 282290 | 229229 | 208208 | 182182 | 273273 |
| G2_U122 | , | 195195 | 151199 | 149125 | 282290 | 229229 | 208208 | 182186 | 273273 |
| G2_U123 | , | 183187 | 171203 | 149149 | 278282 | 229229 | 208208 | 174182 | 273273 |
| G2_U124 | , | 187187 | 159159 | 133157 | 282282 | 229229 | 208208 | 182190 | 277289 |
| G2_U125 | , | 195195 | 171199 | 133157 | 278278 | 229229 | 208208 | 174182 | 289297 |
| G2_U126 | , | 183183 | 171195 | 149149 | 282290 | 229229 | 208208 | 182190 | 289289 |
| G2_U127 | , | 167183 | 171171 | 149157 | 282290 | 229229 | 224228 | 174182 | 273277 |
| G2_U128 | , | 167179 | 151203 | 149157 | 282282 | 229229 | 208208 | 174182 | 273273 |
| G2_U129 | , | 183187 | 171199 | 129149 | 282290 | 229229 | 208208 | 174190 | 273273 |
| G2_U130 | , | 167187 | 151203 | 133133 | 282290 | 229229 | 208208 | 174182 | 273273 |
| G2_U131 | , | 167175 | 167199 | 149157 | 282282 | 229229 | 208208 | 174182 | 273277 |
| G2_U132 | , | 183183 | 171199 | 125133 | 290290 | 229229 | 224228 | 182182 | 273273 |
| G2_U133 | , | 179195 | 151199 | 129133 | 282290 | 229229 | 208224 | 182182 | 273285 |
| G2_U134 | , | 183183 | 195203 | 129149 | 282290 | 229229 | 208208 | 182190 | 273293 |
| G2_U135 | , | 183195 | 151203 | 149149 | 282290 | 229229 | 208208 | 182182 | 273273 |
| Pop |  |  |  |  |  |  |  |  |  |
| G3_U100 | , | 179187 | 171175 | 129153 | 278286 | 229229 | 220224 | 182182 | 273289 |
| G3_U101 | , | 175183 | 175215 | 121129 | 274278 | 229229 | 220232 | 178178 | 285293 |
| G3_U102 | , | 159187 | 163195 | 129129 | 278278 | 229229 | 220228 | 178182 | 277285 |
| G3_U103 | , | 183195 | 163211 | 125133 | 278286 | 229229 | 220220 | 178178 | 277277 |
| G3_U104 | , | 143155 | 175195 | 125129 | 282286 | 229229 | 212220 | 178190 | 273277 |
| G3_U105 | , | 183199 | 175175 | 129137 | 278282 | 229229 | 224232 | 178186 | 277285 |
| G3_U106 | , | 167183 | 179211 | 129145 | 282290 | 229229 | 232232 | 178178 | 277277 |
| G3_U107 | , | 183187 | 175219 | 121133 | 278278 | 229229 | 208220 | 178182 | 273277 |
| G3_U108 | , | 147183 | 199211 | 125169 | 286286 | 229229 | 224224 | 182186 | 289293 |
| G3_U109 | , | 171183 | 207207 | 149169 | 278286 | 229229 | 220228 | 182182 | 277285 |
| G3_U110 | , | 143183 | 167175 | 129169 | 278278 | 229229 | 220220 | 178178 | 273277 |
| G3_U111 | , | 175195 | 175187 | 129149 | 274294 | 229229 | 208220 | 182186 | 265265 |
| G3_U112 | , | 159167 | 175195 | 129137 | 278286 | 229229 | 220228 | 178178 | 273289 |
| G3_U113 | , | 171179 | 167207 | 121129 | 286290 | 229229 | 216228 | 174178 | 277277 |
| G3_U114 | , | 183183 | 211211 | 121137 | 278290 | 229229 | 224228 | 178182 | 285289 |
| Pop |  |  |  |  |  |  |  |  |  |
| G4_U60 | , | 167187 | 175203 | 133145 | 282290 | 229229 | 224224 | 178182 | 281293 |
| G4_U61 | , | 191215 | 179199 | 133153 | 282286 | 229229 | 204232 | 182182 | 281285 |
| G4_U62 | , | 167195 | 199211 | 149149 | 278286 | 229229 | 212228 | 174182 | 281281 |
| G4_U63 | , | 191195 | 203207 | 129149 | 286290 | 229237 | 220232 | 178186 | 289329 |
| G4_U64 | , | 167187 | 163211 | 125153 | 274282 | 237237 | 220236 | 186186 | 289289 |
| G4_U65 | , | 151187 | 203215 | 125129 | 286290 | 229237 | 216224 | 178182 | 281289 |
| G4_U66 | , | 143163 | 175199 | 133149 | 278282 | 229229 | 220228 | 182186 | 289293 |
| G4_U67 | , | 151195 | 175215 | 129129 | 286290 | 229237 | 224232 | 182186 | 285293 |
| G4_U68 | , | 151159 | 175175 | 129153 | 282290 | 229229 | 224228 | 178178 | 285289 |
| G4_U69 | , | 151179 | 163175 | 125129 | 282282 | 229237 | 216224 | 178182 | 281281 |
| G4_U70 | , | 143195 | 151211 | 121121 | 278282 | 229237 | 208208 | 182194 | 277285 |
| G4_U71 | , | 179191 | 175199 | 129169 | 282290 | 229229 | 216220 | 182186 | 289293 |
| G4_U72 | , | 183187 | 191199 | 165169 | 278282 | 229229 | 232232 | 182186 | 285285 |
| G4_U73 | , | 159163 | 179207 | 133133 | 290298 | 237237 | 228232 | 178182 | 281285 |
| G4_U74 | , | 183187 | 199203 | 125129 | 278286 | 229237 | 216224 | 178182 | 277289 |
| Pop |  |  |  |  |  |  |  |  |  |
| G5_U49 | , | 163187 | 155207 | 137153 | 278286 | 229237 | 220228 | 178186 | 281285 |
| G5_U50 | , | 187187 | 199203 | 133145 | 282282 | 229237 | 216236 | 178182 | 285289 |
| G5_U51 | , | 183183 | 175175 | 129149 | 282286 | 229237 | 216216 | 186194 | 281281 |
| G5_U52 | , | 131147 | 175187 | 149161 | 286290 | 229229 | 216220 | 186186 | 285285 |
| G5_U53 | , | 191215 | 171203 | 121149 | 298298 | 229237 | 216224 | 182186 | 277329 |
| G5_U54 | , | 183195 | 151183 | 129161 | 282290 | 229229 | 216224 | 178182 | 289289 |
| G5_U55 | , | 187195 | 175207 | 133169 | 282282 | 229229 | 208224 | 178182 | 277289 |
| G5_U56 | , | 187187 | 203215 | 125133 | 290298 | 229237 | 228232 | 182182 | 289289 |
| G5_U57 | , | 127143 | 191203 | 121153 | 278290 | 229237 | 216224 | 178182 | 281285 |
| G5_U58 | , | 187191 | 195203 | 133169 | 278278 | 229229 | 224236 | 178186 | 277277 |
| G5_U59 | , | 187195 | 175215 | 129141 | 278282 | 229229 | 212224 | 178182 | 285289 |
| Pop |  |  |  |  |  |  |  |  |  |
| H1_P258 | , | 163167 | 155183 | 141141 | 282302 | 229229 | 208232 | 174174 | 281281 |
| H1_P259 | , | 167199 | 155207 | 129141 | 294302 | 229229 | 208208 | 174174 | 281297 |
| H1_P260 | , | 203203 | 187203 | 129141 | 294294 | 229229 | 208208 | 174174 | 297297 |
| H1_P261 | , | 199203 | 203211 | 129141 | 294294 | 229229 | 208208 | 178178 | 281281 |
| H1_P262 | , | 199199 | 207207 | 141141 | 294294 | 229229 | 208208 | 174178 | 281281 |
| H1_P263 | , | 167203 | 195203 | 129141 | 282302 | 229229 | 208208 | 174174 | 281293 |
| H1_P264 | , | 167199 | 183207 | 141141 | 294306 | 229229 | 208232 | 174174 | 281281 |
| H1_P265 | , | 163167 | 159207 | 129141 | 294302 | 229229 | 208208 | 174174 | 281281 |
| H1_P266 | , | 171203 | 207207 | 129129 | 294294 | 229229 | 208208 | 174178 | 281293 |
| H1_P267 | , | 159171 | 155207 | 133141 | 294306 | 229229 | 232232 | 174178 | 281293 |
| H1_P268 | , | 163163 | 155207 | 129141 | 282294 | 229229 | 232232 | 174174 | 293293 |
| H1_P269 | , | 171203 | 155203 | 141141 | 294302 | 229229 | 208212 | 174174 | 293293 |
| H1_P270 | , | 163167 | 155163 | 129141 | 294298 | 229229 | 208212 | 178178 | 281297 |
| H1_P271 | , | 163199 | 155187 | 141141 | 282294 | 229229 | 208216 | 174174 | 281297 |
| H1_P272 | , | 167167 | 155171 | 133141 | 294294 | 229229 | 208208 | 174178 | 281293 |
| Pop |  |  |  |  |  |  |  |  |  |
| H2_P277 | , | 183183 | 195199 | 137141 | 294294 | 229229 | 224224 | 178186 | 297309 |
| H2_P278 | , | 171175 | 199207 | 133133 | 298302 | 229229 | 216224 | 174178 | 273293 |
| H2_P279 | , | 175179 | 195199 | 137141 | 298298 | 229229 | 220224 | 178186 | 273305 |
| H2_P280 | , | 175195 | 191199 | 141145 | 298298 | 229229 | 212224 | 178186 | 305309 |
| H2_P281 | , | 171175 | 195199 | 133141 | 294298 | 229229 | 220224 | 178178 | 269309 |
| H2_P282 | , | 167175 | 163203 | 133137 | 298302 | 229229 | 212224 | 178186 | 285305 |
| H2_P283 | , | 155175 | 195207 | 129145 | 290298 | 229229 | 224224 | 174186 | 269269 |
| H2_P284 | , | 171175 | 191191 | 141141 | 290302 | 229229 | 224224 | 186186 | 305305 |
| H2_P285 | , | 167171 | 199199 | 129141 | 298298 | 229229 | 216224 | 178186 | 277289 |
| H2_P286 | , | 175183 | 183191 | 141141 | 298298 | 229229 | 224224 | 186186 | 273309 |
