# Supplementary material for: Population Genetic Patterns of Threatened European Mudminnow (Umbra krameri Walbaum, 1792) in a Fragmented Landscape: Implications for Conservation Management
Source: PLoS One. 2015 Sep 22;10(9):e0138640. doi: 10.1371/journal.pone.0138640 (PMC4578892; doi:10.1371/journal.pone.0138640)
Supplement: S3 Table — (DOCX) [file pone.0138640.s003.docx]

S3 Table

| **pop** | **Locus** | **Null Present** | **Oosterhout** | **Chakraborty** | **Brookfield 1** | **Brookfield 2** |
| --- | --- | --- | --- | --- | --- | --- |
| **A1** | UkrTet1 | no | -0.0335 | -0.0307 | -0.0282 | 0 |
|  | UkrTet3 | no | -0.0651 | -0.058 | -0.058 | 0 |
|  | UkrTet4 | no | -0.0424 | -0.04 | -0.0385 | 0 |
|  | UkrTet5 | no | -0.026 | -0.0267 | -0.0234 | 0 |
|  | UkrTet6 | no | 0.0232 | 0.0244 | 0.013 | 0.013 |
|  | UkrTet7 | no | -0.013 | -0.006 | -0.0055 | 0 |
|  | UkrTet8 | no | -0.0494 | -0.0544 | -0.0421 | 0 |
|  | UkrTet9 | no | 0.0901 | 0.0903 | 0.072 | 0.072 |
|  | **No loci show evidence for a null allele.** | | | | | |
|  | **This population is probably in Hardy Weinberg equilibrium.** | | | | | |
|  |  |  |  |  |  |  |
|  |  |  |  |  |  |  |
| **pop** | **Locus** | **Null Present** | **Oosterhout** | **Chakraborty** | **Brookfield 1** | **Brookfield 2** |
| **A2** | UkrTet1 | no | -0.084 | -0.0602 | -0.0575 | 0 |
|  | UkrTet3 | no | -0.0597 | -0.0549 | -0.0549 | 0 |
|  | UkrTet4 | no | -0.0717 | -0.0619 | -0.056 | 0 |
|  | UkrTet5 | no | -0.1251 | -0.0977 | -0.0931 | 0 |
|  | UkrTet6 | no | -0.0426 | -0.0213 | -0.0032 | 0 |
|  | UkrTet7 | no | 0.0037 | 0 | 0 | 0 |
|  | UkrTet8 | no | 0.1302 | 0.1515 | 0.1163 | 0.1163 |
|  | UkrTet9 | no | 0.0066 | 0.0182 | 0.0156 | 0.0156 |
|  | **No loci show evidence for a null allele.** | | | | | |
|  | **This population is probably in Hardy Weinberg equilibrium.** | | | | | |
|  |  |  |  |  |  |  |
|  |  |  |  |  |  |  |
| **pop** | **Locus** | **Null Present** | **Oosterhout** | **Chakraborty** | **Brookfield 1** | **Brookfield 2** |
| **A3** | UkrTet1 | no | -0.1351 | -0.1111 | -0.1111 | 0 |
|  | UkrTet3 | no | -0.237 | -0.1628 | -0.1628 | 0 |
|  | UkrTet4 | no | 0.0395 | 0.0323 | 0.0244 | 0.0244 |
|  | UkrTet5 | no | -0.4365 | -0.1594 | -0.1392 | 0 |
|  | UkrTet6 | no | -0.2254 | -0.1111 | -0.0606 | 0 |
|  | UkrTet7 | no | -0.0661 | -0.0667 | -0.0588 | 0 |
|  | UkrTet8 | no | -0.2513 | -0.1765 | -0.1765 | 0 |
|  | UkrTet9 | no | -0.0842 | -0.0753 | -0.0753 | 0 |
|  | **No loci show evidence for a null allele.** | | | | | |
|  | **This population is probably in Hardy Weinberg equilibrium.** | | | | | |
|  |  |  |  |  |  |  |
|  |  |  |  |  |  |  |
| **pop** | **Locus** | **Null Present** | **Oosterhout** | **Chakraborty** | **Brookfield 1** | **Brookfield 2** |
| **A4** | UkrTet1 | no | -0.0091 | -0.0117 | -0.0108 | 0 |
|  | UkrTet3 | no | -0.1234 | -0.0989 | -0.0989 | 0 |
|  | UkrTet4 | no | 0.0871 | 0.1225 | 0.0885 | 0.0885 |
|  | UkrTet5 | no | -0.0525 | -0.0493 | -0.0414 | 0 |
|  | UkrTet6 | no | -0.1437 | -0.0714 | -0.0289 | 0 |
|  | UkrTet7 | no | 0.0568 | 0.0506 | 0.0409 | 0.0409 |
|  | UkrTet8 | no | 0.0476 | 0.0536 | 0.0434 | 0.0434 |
|  | UkrTet9 | no | -0.0456 | -0.0422 | -0.0407 | 0 |
|  | **No loci show evidence for a null allele.** | | | | | |
|  | **This population is probably in Hardy Weinberg equilibrium.** | | |  |  |  |
|  |  |  |  |  |  |  |
|  |  |  |  |  |  |  |
| **pop** | **Locus** | **Null Present** | **Oosterhout** | **Chakraborty** | **Brookfield 1** | **Brookfield 2** |
| **B1** | UkrTet1 | no | -0.0611 | -0.0553 | -0.0533 | 0 |
|  | UkrTet3 | no | -0.1272 | -0.1016 | -0.1016 | 0 |
|  | UkrTet4 | no | 0.0637 | 0.0652 | 0.0557 | 0.0557 |
|  | UkrTet5 | no | -0.0678 | -0.0593 | -0.0571 | 0 |
|  | UkrTet6 | no | -0.0691 | -0.0345 | -0.0079 | 0 |
|  | UkrTet7 | no | 0.0071 | 0.0076 | 0.0071 | 0.0071 |
|  | UkrTet8 | no | 0.0066 | 0.0076 | 0.0071 | 0.0071 |
|  | UkrTet9 | no | -0.0632 | -0.054 | -0.052 | 0 |
|  | **No loci show evidence for a null allele.** | | | | | |
|  | **This population is probably in Hardy Weinberg equilibrium.** | | | | | |
|  |  |  |  |  |  |  |
|  |  |  |  |  |  |  |
| **pop** | **Locus** | **Null Present** | **Oosterhout** | **Chakraborty** | **Brookfield 1** | **Brookfield 2** |
| **B2** | UkrTet1 | no | -0.0481 | -0.0453 | -0.0453 | 0 |
|  | UkrTet3 | no | 0.0274 | 0.0274 | 0.0255 | 0.0255 |
|  | UkrTet4 | **yes** | 0.1447 | 0.1641 | 0.1283 | 0.1283 |
|  | UkrTet5 | no | -0.0246 | -0.0156 | -0.0145 | 0 |
|  | UkrTet6 | no | 0.1891 | 0.4595 | 0.096 | 0.096 |
|  | UkrTet7 | no | -0.0139 | -0.013 | -0.012 | 0 |
|  | UkrTet8 | no | -0.0478 | -0.0461 | -0.0444 | 0 |
|  | UkrTet9 | no | 0.0066 | 0.0038 | 0.0036 | 0.0036 |
|  | **One locus shows evidence for a null allele.** | | | | | |
|  | **This population is possibly in Hardy Weinberg equilibrium with locus UkrTet4, showing signs of a null allele.** | | | | | |
|  |  |  |  |  |  |  |
|  |  |  |  |  |  |  |
| **pop** | **Locus** | **Null Present** | **Oosterhout** | **Chakraborty** | **Brookfield 1** | **Brookfield 2** |
| **B3** | UkrTet1 | **yes** | 0.1435 | 0.1724 | 0.1351 | 0.1351 |
|  | UkrTet3 | no | 0.035 | 0.0448 | 0.04 | 0.04 |
|  | UkrTet4 | no | -0.1577 | -0.1204 | -0.1204 | 0 |
|  | UkrTet5 | no | -0.1457 | -0.115 | -0.1009 | 0 |
|  | UkrTet6 | no | -0.1056 | -0.0526 | -0.0169 | 0 |
|  | UkrTet7 | no | -0.1505 | -0.1142 | -0.1142 | 0 |
|  | UkrTet8 | no | 0.1776 | 0.1935 | 0.1379 | 0.1379 |
|  | UkrTet9 | no | -0.0654 | -0.0667 | -0.0588 | 0 |
|  | **One locus shows evidence for a null allele.** | | | | | |
|  | **This population is possibly in Hardy Weinberg equilibrium with locus UkrTet1, showing signs of a null allele.** | | | | | |
|  |  |  |  |  |  |  |
|  |  |  |  |  |  |  |
| **pop** | **Locus** | **Null Present** | **Oosterhout** | **Chakraborty** | **Brookfield 1** | **Brookfield 2** |
| **C1** | UkrTet1 | no | -0.1056 | -0.0526 | -0.0169 | 0 |
|  | UkrTet3 | no | -0.0152 | -0.0256 | -0.0227 | 0 |
|  | UkrTet4 | no | -0.1457 | -0.1111 | -0.0976 | 0 |
|  | UkrTet5 | no | -0.1457 | -0.1111 | -0.0976 | 0 |
|  | UkrTet6 | no | 0 | 0 | 0 | 0 |
|  | UkrTet7 | no | -0.0036 | 0.0164 | 0.0123 | 0.0123 |
|  | UkrTet8 | no | -0.331 | -0.1321 | -0.0959 | 0 |
|  | UkrTet9 | no | -0.1457 | -0.1268 | -0.1111 | 0 |
|  | **No loci show evidence for a null allele.** | | | | | |
|  | **This population is probably in Hardy Weinberg equilibrium.** | | | | |  |
|  |  |  |  |  |  |  |
|  |  |  |  |  |  |  |
| **pop** | **Locus** | **Null Present** | **Oosterhout** | **Chakraborty** | **Brookfield 1** | **Brookfield 2** |
| **C2** | UkrTet1 | no | -0.0249 | -0.037 | -0.0233 | 0 |
|  | UkrTet3 | no | -0.0664 | -0.0627 | -0.0579 | 0 |
|  | UkrTet4 | no | 0.038 | 0.0646 | 0.0421 | 0.0421 |
|  | UkrTet5 | no | -0.0445 | -0.0425 | -0.0315 | 0 |
|  | UkrTet6 | no | 0 | 0 | 0 | 0 |
|  | UkrTet7 | no | -0.0091 | -0.0189 | -0.0141 | 0 |
|  | UkrTet8 | no | -0.1056 | -0.0526 | -0.0169 | 0 |
|  | UkrTet9 | no | -0.0156 | -0.0204 | -0.0163 | 0 |
|  | **No loci show evidence for a null allele.** | | | | |  |
|  | **This population is probably in Hardy Weinberg equilibrium.** | | | | | |
|  |  |  |  |  |  |  |
|  |  |  |  |  |  |  |
| **pop** | **Locus** | **Null Present** | **Oosterhout** | **Chakraborty** | **Brookfield 1** | **Brookfield 2** |
| **D1** | UkrTet1 | no | -0.0575 | -0.0526 | -0.036 | 0 |
|  | UkrTet3 | no | 0.0213 | 0.0323 | 0.0244 | 0.0244 |
|  | UkrTet4 | no | 0.0937 | 0.1089 | 0.0653 | 0.0653 |
|  | UkrTet5 | no | -0.0219 | -0.0248 | -0.0209 | 0 |
|  | UkrTet6 | no | 0 | 0 | 0 | 0 |
|  | UkrTet7 | no | 0.1162 | 0.1589 | 0.0974 | 0.0974 |
|  | UkrTet8 | no | -0.0575 | -0.0594 | -0.0498 | 0 |
|  | UkrTet9 | no | -0.0445 | -0.0019 | -0.0014 | 0 |
|  | **No loci show evidence for a null allele.** | | | | | |
|  | **This population is probably in Hardy Weinberg equilibrium.** | | | | | |
|  |  |  |  |  |  |  |
|  |  |  |  |  |  |  |
| **pop** | **Locus** | **Null Present** | **Oosterhout** | **Chakraborty** | **Brookfield 1** | **Brookfield 2** |
| **D2** | UkrTet1 | **yes** | 0.2531 | 0.3651 | 0.2097 | 0.2097 |
|  | UkrTet3 | no | 0.0538 | 0.0184 | 0.0106 | 0.0106 |
|  | UkrTet4 | no | -0.0793 | -0.0811 | -0.0596 | 0 |
|  | UkrTet5 | no | 0.108 | 0.0698 | 0.0335 | 0.0335 |
|  | UkrTet6 | no | -0.0513 | -0.0256 | -0.0046 | 0 |
|  | UkrTet7 | no | -0.1569 | -0.0619 | -0.0277 | 0 |
|  | UkrTet8 | no | -0.2767 | -0.1842 | -0.1728 | 0 |
|  | UkrTet9 | no | 0.0534 | 0.0438 | 0.0332 | 0.0332 |
|  | **One locus shows evidence for a null allele.** | | |  |  |  |
|  | **This population is possibly in Hardy Weinberg equilibrium with locus UkrTet1, showing signs of a null allele.** | | | | | |
|  |  |  |  |  |  |  |
|  |  |  |  |  |  |  |
| **pop** | **Locus** | **Null Present** | **Oosterhout** | **Chakraborty** | **Brookfield 1** | **Brookfield 2** |
| **D3** | UkrTet1 | no | -0.1687 | -0.1111 | -0.0976 | 0 |
|  | UkrTet3 | no | 0.0456 | 0.0361 | 0.0274 | 0.0274 |
|  | UkrTet4 | no | 0.1254 | 0.1266 | 0.0881 | 0.0881 |
|  | UkrTet5 | no | 0.108 | 0.0698 | 0.0335 | 0.0335 |
|  | UkrTet6 | no | 0 | 0 | 0 | 0 |
|  | UkrTet7 | no | -0.2082 | -0.0738 | -0.0409 | 0 |
|  | UkrTet8 | no | 0.0129 | 0.0036 | 0.0029 | 0.0029 |
|  | UkrTet9 | no | 0.0706 | 0.0698 | 0.0533 | 0.0533 |
|  | **No loci show evidence for a null allele.** | | | | | |
|  | **This population is probably in Hardy Weinberg equilibrium.** | | | | | |
|  |  |  |  |  |  |  |
|  |  |  |  |  |  |  |
| **pop** | **Locus** | **Null Present** | **Oosterhout** | **Chakraborty** | **Brookfield 1** | **Brookfield 2** |
| **D4** | UkrTet1 | no | -0.0535 | -0.0375 | -0.0354 | 0 |
|  | UkrTet3 | no | -0.0389 | -0.0405 | -0.0383 | 0 |
|  | UkrTet4 | no | -0.1045 | -0.0899 | -0.0899 | 0 |
|  | UkrTet5 | no | -0.0779 | -0.0714 | -0.0674 | 0 |
|  | UkrTet6 | no | -0.0513 | -0.0256 | -0.0046 | 0 |
|  | UkrTet7 | no | -0.036 | -0.0375 | -0.0354 | 0 |
|  | UkrTet8 | no | -0.0158 | -0.0256 | -0.0227 | 0 |
|  | UkrTet9 | no | 0.0208 | 0.0214 | 0.0191 | 0.0191 |
|  | **No loci show evidence for a null allele.** | | | | | |
|  | **This population is probably in Hardy Weinberg equilibrium.** | | | | | |
|  |  |  |  |  |  |  |
|  |  |  |  |  |  |  |
| **pop** | **Locus** | **Null Present** | **Oosterhout** | **Chakraborty** | **Brookfield 1** | **Brookfield 2** |
| **D5** | UkrTet1 | no | 0.0498 | 0.0476 | 0.0426 | 0.0426 |
|  | UkrTet3 | no | -0.2147 | -0.1494 | -0.1494 | 0 |
|  | UkrTet4 | no | -0.0661 | -0.0667 | -0.0588 | 0 |
|  | UkrTet5 | no | 0.1959 | 0.2308 | 0.1463 | 0.1463 |
|  | UkrTet6 | no | 0 | 0 | 0 | 0 |
|  | UkrTet7 | no | 0.0761 | 0.0476 | 0.0361 | 0.0361 |
|  | UkrTet8 | no | -0.1092 | -0.0667 | -0.0588 | 0 |
|  | UkrTet9 | no | -0.0295 | -0.039 | -0.0345 | 0 |
|  | **No loci show evidence for a null allele.** | | | | | |
|  | **This population is probably in Hardy Weinberg equilibrium.** | | | | | |
|  |  |  |  |  |  |  |
|  |  |  |  |  |  |  |
| **pop** | **Locus** | **Null Present** | **Oosterhout** | **Chakraborty** | **Brookfield 1** | **Brookfield 2** |
| **E1** | UkrTet1 | no | 0.119 | 0.1249 | 0.0967 | 0.0967 |
|  | UkrTet3 | no | 0.012 | 0.0102 | 0.0094 | 0.0094 |
|  | UkrTet4 | no | -0.0303 | -0.0369 | -0.0288 | 0 |
|  | UkrTet5 | no | 0.0298 | 0.0323 | 0.0152 | 0.0152 |
|  | UkrTet6 | no | -0.0253 | -0.0127 | -0.0012 | 0 |
|  | UkrTet7 | no | 0.0254 | 0.0179 | 0.0142 | 0.0142 |
|  | UkrTet8 | no | 0.108 | 0.1549 | 0.078 | 0.078 |
|  | UkrTet9 | no | -0.0211 | -0.0216 | -0.0169 | 0 |
|  | **No loci show evidence for a null allele.** | | | | | |
|  | **This population is probably in Hardy Weinberg equilibrium.** | | | | | |
|  |  |  |  |  |  |  |
|  |  |  |  |  |  |  |
| **pop** | **Locus** | **Null Present** | **Oosterhout** | **Chakraborty** | **Brookfield 1** | **Brookfield 2** |
| **E2** | UkrTet1 | no | -0.0266 | -0.0224 | -0.0198 | 0 |
|  | UkrTet3 | **yes** | 0.1065 | 0.1167 | 0.0981 | 0.0981 |
|  | UkrTet4 | no | 0.1524 | 0.1561 | 0.1098 | 0.1098 |
|  | UkrTet5 | no | 0.0233 | 0.0244 | 0.0141 | 0.0141 |
|  | UkrTet6 | no | -0.0345 | -0.0332 | -0.0272 | 0 |
|  | UkrTet7 | no | 0.0193 | 0.0244 | 0.0202 | 0.0202 |
|  | UkrTet8 | no | -0.3267 | -0.1268 | -0.0922 | 0 |
|  | UkrTet9 | no | -0.0087 | -0.0294 | -0.0241 | 0 |
|  | **One locus shows evidence for a null allele.** | | | | | |
|  | **This population is possibly in Hardy Weinberg equilibrium with locus UkrTet3, showing signs of a null allele.** | | | | | |
|  |  |  |  |  |  |  |
|  |  |  |  |  |  |  |
| **pop** | **locus** | **Null Present** | **Oosterhout** | **Chakraborty** | **Brookfield 1** | **Brookfield 2** |
| **E3** | UkrTet1 | **yes** | 0.1646 | 0.1987 | 0.1471 | 0.1471 |
|  | UkrTet3 | no | 0.0066 | 0.0051 | 0.0047 | 0.0047 |
|  | UkrTet4 | no | 0.042 | 0.0288 | 0.0217 | 0.0217 |
|  | UkrTet5 | no | 0.0747 | 0.0706 | 0.0539 | 0.0539 |
|  | UkrTet6 | no | -0.2127 | -0.0843 | -0.0465 | 0 |
|  | UkrTet7 | no | -0.0169 | -0.0213 | -0.0189 | 0 |
|  | UkrTet8 | no | -0.2254 | -0.1111 | -0.0606 | 0 |
|  | UkrTet9 | no | -0.0177 | -0.0198 | -0.0176 | 0 |
|  | **One locus shows evidence for a null allele.** | | | | |  |
|  | **This population is possibly in Hardy Weinberg equilibrium with locus UkrTet1, showing signs of a null allele.** | | | | | |
|  |  |  |  |  |  |  |
|  |  |  |  |  |  |  |
| **pop** | **Locus** | **Null Present** | **Oosterhout** | **Chakraborty** | **Brookfield 1** | **Brookfield 2** |
| **E4** | UkrTet1 | no | -0.0571 | -0.0526 | -0.0507 | 0 |
|  | UkrTet3 | no | -0.0333 | -0.0332 | -0.032 | 0 |
|  | UkrTet4 | no | 0.0964 | 0.0909 | 0.0698 | 0.0698 |
|  | UkrTet5 | no | -0.0402 | -0.0405 | -0.0358 | 0 |
|  | UkrTet6 | no | -0.1746 | -0.0676 | -0.0327 | 0 |
|  | UkrTet7 | no | -0.0585 | -0.0569 | -0.0526 | 0 |
|  | UkrTet8 | no | -0.2932 | -0.1823 | -0.1588 | 0 |
|  | UkrTet9 | no | 0.0106 | 0.0069 | 0.0061 | 0.0061 |
|  | **No loci show evidence for a null allele.** | | | | | |
|  | **This population is probably in Hardy Weinberg equilibrium.** | | | | | |
|  |  |  |  |  |  |  |
|  |  |  |  |  |  |  |
| **pop** | **Locus** | **Null Present** | **Oosterhout** | **Chakraborty** | **Brookfield 1** | **Brookfield 2** |
| **E5** | UkrTet1 | no | 0.0071 | 0.0123 | 0.011 | 0.011 |
|  | UkrTet3 | no | 0.0107 | 0.0323 | 0.0244 | 0.0244 |
|  | UkrTet4 | no | -0.4223 | -0.1429 | -0.125 | 0 |
|  | UkrTet5 | no | -0.4731 | -0.194 | -0.1688 | 0 |
|  | UkrTet6 | no | -0.3167 | -0.1111 | -0.0811 | 0 |
|  | UkrTet7 | no | -0.4365 | -0.1594 | -0.1392 | 0 |
|  | UkrTet8 | no | 0.1593 | 0.1837 | 0.1139 | 0.1139 |
|  | UkrTet9 | no | -0.2005 | -0.1364 | -0.1364 | 0 |
|  | **No loci show evidence for a null allele.** | | | | | |
|  | **This population is probably in Hardy Weinberg equilibrium.** | | | | | |
|  |  |  |  |  |  |  |
|  |  |  |  |  |  |  |
| **pop** | **Locus** | **Null Present** | **Oosterhout** | **Chakraborty** | **Brookfield 1** | **Brookfield 2** |
| **E6** | UkrTet1 | no | 0.0248 | 0.0184 | 0.0168 | 0.0168 |
|  | UkrTet3 | no | -0.0768 | -0.0686 | -0.0686 | 0 |
|  | UkrTet4 | no | -0.052 | -0.0549 | -0.0497 | 0 |
|  | UkrTet5 | no | -0.1221 | -0.0884 | -0.0798 | 0 |
|  | UkrTet6 | no | 0.1761 | 0.2233 | 0.1447 | 0.1447 |
|  | UkrTet7 | no | 0.0335 | 0.0357 | 0.0308 | 0.0308 |
|  | UkrTet8 | no | -0.0478 | -0.046 | -0.0392 | 0 |
|  | UkrTet9 | no | -0.0479 | -0.0435 | -0.0415 | 0 |
|  | **No loci show evidence for a null allele.** | | | | | |
|  | **This population is probably in Hardy Weinberg equilibrium.** | | | | | |
|  |  |  |  |  |  |  |
|  |  |  |  |  |  |  |
| **pop** | **Locus** | **Null Present** | **Oosterhout** | **Chakraborty** | **Brookfield 1** | **Brookfield 2** |
| **E7** | UkrTet1 | no | 0.0185 | 0.0177 | 0.0158 | 0.0158 |
|  | UkrTet3 | no | -0.0047 | -0.0026 | -0.0024 | 0 |
|  | UkrTet4 | no | -0.1548 | -0.1208 | -0.1208 | 0 |
|  | UkrTet5 | no | 0.0137 | 0.0105 | 0.0089 | 0.0089 |
|  | UkrTet6 | no | -0.0101 | -0.0412 | -0.0283 | 0 |
|  | UkrTet7 | no | -0.013 | -0.0141 | -0.0125 | 0 |
|  | UkrTet8 | no | 0.0248 | -0.0017 | -0.0013 | 0 |
|  | UkrTet9 | no | -0.1488 | -0.1096 | -0.1055 | 0 |
|  | **No loci show evidence for a null allele.** | | | | | |
|  | **This population is probably in Hardy Weinberg equilibrium.** | | | | | |
|  |  |  |  |  |  |  |
|  |  |  |  |  |  |  |
| **pop** | **Locus** | **Null Present** | **Oosterhout** | **Chakraborty** | **Brookfield 1** | **Brookfield 2** |
| **E8** | UkrTet1 | no | -0.028 | 0.005 | 0.0033 | 0.0033 |
|  | UkrTet3 | no | -0.0513 | -0.0471 | -0.0309 | 0 |
|  | UkrTet4 | no | -0.1633 | -0.0811 | -0.0359 | 0 |
|  | UkrTet5 | no | -0.1874 | -0.1502 | -0.1411 | 0 |
|  | UkrTet6 | no | -1 | -0.3333 | -0.3333 | 0 |
|  | UkrTet7 | no | -0.5929 | -0.2346 | -0.2346 | 0 |
|  | UkrTet8 | no | -0.1569 | -0.0619 | -0.0277 | 0 |
|  | UkrTet9 | no | -0.5696 | -0.2158 | -0.2158 | 0 |
|  | **No loci show evidence for a null allele.** | | | | | |
|  | **This population is probably in Hardy Weinberg equilibrium.** | | | | | |
|  |  |  |  |  |  |  |
|  |  |  |  |  |  |  |
| **pop** | **Locus** | **Null Present** | **Oosterhout** | **Chakraborty** | **Brookfield 1** | **Brookfield 2** |
| **F1** | UkrTet1 | **yes** | 0.1854 | 0.2308 | 0.1727 | 0.1727 |
|  | UkrTet3 | no | -0.0623 | -0.0563 | -0.0563 | 0 |
|  | UkrTet4 | no | 0.0053 | -0.0107 | -0.0091 | 0 |
|  | UkrTet5 | no | 0.0483 | 0.0566 | 0.0458 | 0.0458 |
|  | UkrTet6 | no | 0.0264 | 0.0141 | 0.009 | 0.009 |
|  | UkrTet7 | no | -0.0647 | -0.051 | -0.0428 | 0 |
|  | UkrTet8 | no | -0.0806 | -0.0835 | -0.0567 | 0 |
|  | UkrTet9 | no | -0.0309 | -0.0331 | -0.0307 | 0 |
|  | **One locus shows evidence for a null allele.** | | | | | |
|  | **This population is possibly in Hardy Weinberg equilibrium with locus UkrTet1, showing signs of a null allele.** | | | | | |
|  |  |  |  |  |  |  |
|  |  |  |  |  |  |  |
| **pop** | **Locus** | **Null Present** | **Oosterhout** | **Chakraborty** | **Brookfield 1** | **Brookfield 2** |
| **F2** | UkrTet1 | no | -0.0807 | -0.0667 | -0.0588 | 0 |
|  | UkrTet3 | no | -0.2147 | -0.1494 | -0.1494 | 0 |
|  | UkrTet4 | no | -0.2513 | -0.1765 | -0.1765 | 0 |
|  | UkrTet5 | no | -0.5564 | -0.2048 | -0.2048 | 0 |
|  | UkrTet6 | no | 0.1816 | 0.2453 | 0.1566 | 0.1566 |
|  | UkrTet7 | no | -0.331 | -0.1321 | -0.0959 | 0 |
|  | UkrTet8 | no | -0.2513 | -0.1765 | -0.1765 | 0 |
|  | UkrTet9 | no | -0.1208 | -0.0989 | -0.0989 | 0 |
|  | **No loci show evidence for a null allele.** | | | | | |
|  | **This population is probably in Hardy Weinberg equilibrium.** | | | | | |
|  |  |  |  |  |  |  |
|  |  |  |  |  |  |  |
| **pop** | **Locus** | **Null Present** | **Oosterhout** | **Chakraborty** | **Brookfield 1** | **Brookfield 2** |
| **F3** | UkrTet1 | no | 0.06 | 0.0588 | 0.0526 | 0.0526 |
|  | UkrTet3 | no | -0.018 | -0.0169 | -0.0164 | 0 |
|  | UkrTet4 | no | -0.0726 | -0.0673 | -0.0649 | 0 |
|  | UkrTet5 | no | 0.0459 | 0.0354 | 0.0285 | 0.0285 |
|  | UkrTet6 | no | -0.2254 | -0.1628 | -0.1261 | 0 |
|  | UkrTet7 | no | -0.0044 | 0.0015 | 0.0013 | 0.0013 |
|  | UkrTet8 | no | -0.1012 | -0.073 | -0.0644 | 0 |
|  | UkrTet9 | no | -0.062 | -0.0563 | -0.0563 | 0 |
|  | **No loci show evidence for a null allele.** | | | | | |
|  | **This population is probably in Hardy Weinberg equilibrium.** | | | | | |
|  |  |  |  |  |  |  |
|  |  |  |  |  |  |  |
| **pop** | **Locus** | **Null Present** | **Oosterhout** | **Chakraborty** | **Brookfield 1** | **Brookfield 2** |
| **F4** | UkrTet1 | no | -0.05 | -0.0435 | -0.0419 | 0 |
|  | UkrTet3 | no | -0.0174 | -0.0223 | -0.0207 | 0 |
|  | UkrTet4 | no | -0.0278 | -0.0169 | -0.015 | 0 |
|  | UkrTet5 | no | 0.1229 | 0.1398 | 0.1016 | 0.1016 |
|  | UkrTet6 | no | 0.0551 | 0.0576 | 0.0438 | 0.0438 |
|  | UkrTet7 | no | -0.1743 | -0.1364 | -0.1364 | 0 |
|  | UkrTet8 | no | -0.0179 | -0.0135 | -0.0108 | 0 |
|  | UkrTet9 | no | -0.0578 | -0.0474 | -0.0457 | 0 |
|  | **No loci show evidence for a null allele.** | | | | | |
|  | **This population is probably in Hardy Weinberg equilibrium.** | | | | | |
|  |  |  |  |  |  |  |
|  |  |  |  |  |  |  |
| **pop** | **Locus** | **Null Present** | **Oosterhout** | **Chakraborty** | **Brookfield 1** | **Brookfield 2** |
| **G1** | UkrTet1 | no | 0.0254 | 0.0257 | 0.0229 | 0.0229 |
|  | UkrTet3 | no | -0.0841 | -0.0742 | -0.0714 | 0 |
|  | UkrTet4 | no | 0.0024 | 0 | 0 | 0 |
|  | UkrTet5 | no | 0.0783 | 0.0994 | 0.0713 | 0.0713 |
|  | UkrTet6 | no | 0 | 0 | 0 | 0 |
|  | UkrTet7 | no | -0.2491 | -0.1732 | -0.1662 | 0 |
|  | UkrTet8 | no | -0.0936 | -0.0746 | -0.0658 | 0 |
|  | UkrTet9 | no | 0.0012 | -0.0014 | -0.0012 | 0 |
|  | **No loci show evidence for a null allele.** | | | | | |
|  | **This population is probably in Hardy Weinberg equilibrium.** | | | | | |
|  |  |  |  |  |  |  |
|  |  |  |  |  |  |  |
| **pop** | **Locus** | **Null Present** | **Oosterhout** | **Chakraborty** | **Brookfield 1** | **Brookfield 2** |
| **G2** | UkrTet1 | no | 0.1167 | 0.1248 | 0.0966 | 0.0966 |
|  | UkrTet3 | no | -0.0409 | -0.0317 | -0.0294 | 0 |
|  | UkrTet4 | no | 0.0357 | 0.0244 | 0.0196 | 0.0196 |
|  | UkrTet5 | no | -0.1266 | -0.0772 | -0.0608 | 0 |
|  | UkrTet6 | no | 0 | 0 | 0 | 0 |
|  | UkrTet7 | no | 0.1905 | 0.1855 | 0.0706 | 0.0706 |
|  | UkrTet8 | no | -0.1891 | -0.1282 | -0.1064 | 0 |
|  | UkrTet9 | no | 0.1343 | 0.1346 | 0.0816 | 0.0816 |
|  | **No loci show evidence for a null allele.** | | | | | |
|  | **This population is probably in Hardy Weinberg equilibrium.** | | | | | |
|  |  |  |  |  |  |  |
|  |  |  |  |  |  |  |
| **pop** | **Locus** | **Null Present** | **Oosterhout** | **Chakraborty** | **Brookfield 1** | **Brookfield 2** |
| **G3** | UkrTet1 | no | -0.0582 | -0.05 | -0.0482 | 0 |
|  | UkrTet3 | no | 0.0233 | 0.0283 | 0.0253 | 0.0253 |
|  | UkrTet4 | no | -0.0889 | -0.0728 | -0.0701 | 0 |
|  | UkrTet5 | no | -0.0065 | -0.0123 | -0.0104 | 0 |
|  | UkrTet6 | no | 0 | 0 | 0 | 0 |
|  | UkrTet7 | no | 0.0078 | 0.0179 | 0.0152 | 0.0152 |
|  | UkrTet8 | no | 0.0733 | 0.0698 | 0.0496 | 0.0496 |
|  | UkrTet9 | no | 0.0188 | 0.0164 | 0.0139 | 0.0139 |
|  | **No loci show evidence for a null allele.** | | | | | |
|  | **This population is probably in Hardy Weinberg equilibrium.** | | | | | |
|  |  |  |  |  |  |  |
|  |  |  |  |  |  |  |
| **pop** | **Locus** | **Null Present** | **Oosterhout** | **Chakraborty** | **Brookfield 1** | **Brookfield 2** |
| **G4** | UkrTet1 | no | -0.0611 | -0.0563 | -0.0563 | 0 |
|  | UkrTet3 | no | -0.044 | -0.0422 | -0.0407 | 0 |
|  | UkrTet4 | no | 0.0611 | 0.0704 | 0.0602 | 0.0602 |
|  | UkrTet5 | no | -0.114 | -0.0995 | -0.0957 | 0 |
|  | UkrTet6 | no | 0.0483 | 0.0526 | 0.0308 | 0.0308 |
|  | UkrTet7 | no | 0.0245 | 0.027 | 0.0241 | 0.0241 |
|  | UkrTet8 | no | -0.1157 | -0.0778 | -0.0686 | 0 |
|  | UkrTet9 | no | 0.0329 | 0.0308 | 0.0262 | 0.0262 |
|  | **No loci show evidence for a null allele.** | | | | | |
|  | **This population is probably in Hardy Weinberg equilibrium.** | | | | | |
|  |  |  |  |  |  |  |
|  |  |  |  |  |  |  |
| **pop** | **Locus** | **Null Present** | **Oosterhout** | **Chakraborty** | **Brookfield 1** | **Brookfield 2** |
| **G5** | UkrTet1 | no | 0.0568 | 0.0538 | 0.0457 | 0.0457 |
|  | UkrTet3 | no | -0.027 | -0.0256 | -0.0244 | 0 |
|  | UkrTet4 | no | -0.0647 | -0.0591 | -0.0591 | 0 |
|  | UkrTet5 | no | 0.0913 | 0.0994 | 0.0791 | 0.0791 |
|  | UkrTet6 | no | -0.3258 | -0.1579 | -0.1065 | 0 |
|  | UkrTet7 | no | -0.0776 | -0.068 | -0.0645 | 0 |
|  | UkrTet8 | no | -0.1019 | -0.0849 | -0.0758 | 0 |
|  | UkrTet9 | no | 0.1362 | 0.1619 | 0.12 | 0.12 |
|  | **No loci show evidence for a null allele.** | | | | |  |
|  | **This population is probably in Hardy Weinberg equilibrium.** | | | | |  |
|  |  |  |  |  |  |  |
|  |  |  |  |  |  |  |
| **pop** | **Locus** | **Null Present** | **Oosterhout** | **Chakraborty** | **Brookfield 1** | **Brookfield 2** |
| **H1** | UkrTet1 | no | 0.0403 | 0.0421 | 0.0358 | 0.0358 |
|  | UkrTet3 | no | -0.0379 | -0.0359 | -0.0332 | 0 |
|  | UkrTet4 | no | -0.1277 | -0.0865 | -0.0635 | 0 |
|  | UkrTet5 | no | -0.065 | -0.0619 | -0.049 | 0 |
|  | UkrTet6 | no | 0 | 0 | 0 | 0 |
|  | UkrTet7 | no | 0.1185 | 0.1643 | 0.0895 | 0.0895 |
|  | UkrTet8 | no | 0.1397 | 0.1892 | 0.0895 | 0.0895 |
|  | UkrTet9 | no | 0.0782 | 0.1083 | 0.0717 | 0.0717 |
|  | **No loci show evidence for a null allele.** | | | | |  |
|  | **This population is probably in Hardy Weinberg equilibrium.** | | | | | |
|  |  |  |  |  |  |  |
|  |  |  |  |  |  |  |
| **pop** | **Locus** | **Null Present** | **Oosterhout** | **Chakraborty** | **Brookfield 1** | **Brookfield 2** |
| **H2** | UkrTet1 | no | -0.1433 | -0.0843 | -0.0795 | 0 |
|  | UkrTet3 | no | -0.0158 | -0.0127 | -0.0112 | 0 |
|  | UkrTet4 | no | 0.0099 | 0.0106 | 0.0087 | 0.0087 |
|  | UkrTet5 | no | 0.0727 | 0.0783 | 0.0536 | 0.0536 |
|  | UkrTet6 | no | 0 | 0 | 0 | 0 |
|  | UkrTet7 | no | -0.3167 | -0.1111 | -0.0811 | 0 |
|  | UkrTet8 | no | -0.1106 | -0.0938 | -0.0759 | 0 |
|  | UkrTet9 | no | 0.0237 | 0.0244 | 0.0217 | 0.0217 |
|  | **No loci show evidence for a null allele.** | | | | | |
|  | **This population is probably in Hardy Weinberg equilibrium.** | | | | | |
